# Increased power by harmonizing structural MRI site differences with the ComBat batch adjustment method in ENIGMA

**DOI:** 10.1016/j.neuroimage.2020.116956

**Published:** 2020-05-26

**Authors:** Joaquim Radua, Eduard Vieta, Russell Shinohara, Peter Kochunov, Yann Quidé, Melissa J. Green, Cynthia S. Weickert, Thomas Weickert, Jason Bruggemann, Tilo Kircher, Igor Nenadić, Murray J. Cairns, Marc Seal, Ulrich Schall, Frans Henskens, Janice M. Fullerton, Bryan Mowry, Christos Pantelis, Rhoshel Lenroot, Vanessa Cropley, Carmel Loughland, Rodney Scott, Daniel Wolf, Theodore D Satterthwaite, Yunlong Tan, Kang Sim, Fabrizio Piras, Gianfranco Spalletta, Nerisa Banaj, Edith Pomarol-Clotet, Aleix Solanes, Anton Albajes-Eizagirre, Erick J. Canales-Rodríguez, Salvador Sarro, Annabella Di Giorgio, Alessandro Bertolino, Michael Stäblein, Viola Oertel, Christian Knöchel, Stefan Borgwardt, Stefan du Plessis, Je-Yeon Yun, Jun Soo Kwon, Udo Dannlowski, Tim Hahn, Dominik Grotegerd, Clara Alloz, Celso Arango, Joost Janssen, Covadonga Díaz-Caneja, Wenhao Jiang, Vince Calhoun, Stefan Ehrlich, Kun Yang, Nicola G. Cascella, Yoichiro Takayanagi, Akira Sawa, Alexander Tomyshev, Irina Lebedeva, Vasily Kaleda, Matthias Kirschner, Cyril Hoschl, David Tomecek, Antonin Skoch, Therese van Amelsvoort, Geor Bakker, Anthony James, Adrian Preda, Andrea Weideman, Dan J. Stein, Fleur Howells, Anne Uhlmann, Henk Temmingh, Carlos López-Jaramillo, Ana Díaz-Zuluaga, Lydia Fortea, Eloy Martinez-Heras, Elisabeth Solana, Sara Llufriu, Neda Jahanshad, Paul Thompson, Jessica Turner, Theo van Erp, ENIGMA Consortium collaborators

**Affiliations:** a Imaging of Mood- and Anxiety-Related Disorders (IMARD) Group, Institut d’Investigacions Biomèdiques August Pi i Sunyer (IDIBAPS), Barcelona, Spain; b CIBERSAM, Madrid, Spain; c Early Psychosis: Interventions and Clinical-detection (EPIC) Lab, Institute of Psychiatry, Psychology and Neuroscience, King’s College London, London, UK; d Department of Clinical Neuroscience, Stockholm Health Care Services, Stockholm County Council, Karolinska Institutet, Stockholm, Sweden; e Bipolar and depressive disorders, Institut d’Investigacions Biomèdiques August Pi i Sunyer (IDIBAPS), Barcelona, Spain; f Barcelona Bipolar Disorders Program, Institute of Neurosciences, Hospital Clinic de Barcelona, Barcelona, Spain; g University of Barcelona, Barcelona, Spain; h Penn Statistics in Imaging and Visualization Center, Department of Biostatistics, Epidemiology, and Informatics, University of Pennsylvania, Philadelphia, PA, USA; i Center for Biomedical Image Computing and Analytics, University of Pennsylvania, Philadelphia, PA, USA; j Maryland Psychiatric Research Center, University of Maryland School of Medicine, Baltimore, MD, USA; k School of Psychiatry, University of New South Wales, Sydney, NSW, Australia; l Neuroscience Research Australia, Sydney, NSW, Australia; m Department of Neuroscience & Physiology, Upstate Medical University, Syracuse, Newyork, NY, USA; n Department of Psychiatry and Psychotherapy, Philipps-University Marburg, Marburg, Germany; o University of Newcastle, Newcastle, NSW, Australia; p Hunter Medical Research Institute, Newcastle, NSW, Australia; q Murdoch Children’s Research Institute, Melbourne, VIC, Australia; r The University of Melbourne, Australia; s Health Behaviour Research Group, School of Medicine and Public Health, University of Newcastle, Newcastle, NSW, Australia; t Queensland Brain Institute, The University of Queensland, Brisbane, QLD, Australia; u Queensland Centre for Mental Health Research, The University of Queensland, Brisbane, QLD, Australia; v Melbourne Neuropsychiatry Centre, Dept. of Psychiatry, University of Melbourne, Melbourne, VIC, Australia; w North Western Mental Health, Melbourne Health, Melbourne, VIC, Australia; x University of New Mexico, Albuquerque, NM, USA; y Department of Psychiatry, University of Pennsylvania, Philadelphia, PA, USA; z Psychiatry Research Center, Beijing Huilongguan Hospital, Beijing, China; aa West Region and Research Division, Institute of Mental Health, Singapore, Singapore; ab Yong Loo Lin School of Medicine, National University of Singapore, Singapore, Singapore; ac Lee Kong Chian School of Medicine, Nanyang Technological University, Singapore, Singapore; ad Laboratory of Neuropsychiatry, Department of Clinical and Behavioral Neurology, IRCCS Santa Lucia Foundation, Rome, Italy; ae Division of Neuropsychiatry, Menninger Department of Psychiatry and Behavioral Sciences, Baylor College of Medicine, Houston, TX, USA; af FIDMAG Germanes Hospitalàries Research Foundation, Barcelona, Spain; ag Department of Psychiatry and Forensic Medicine, School of Medicine, Autonomous University of Barcelona, Barcelona, Spain; ah Department of Radiology, Centre Hospitalier Universitaire Vaudois (CHUV), Lausanne, Switzerland; ai Signal Processing Lab (LTS5), École Polytechnique Fédérale de Lausanne, Lausanne, Switzerland; aj School of Medicine, Universitat Internacional de Catalunya, Barcelona, Spain; ak IRCCS Casa Sollievo della Sofferenza, San Giovanni Rotondo, Italy; al Department of Basic Medical Science, Neuroscience and Sense Organs, University of Bari ‘Aldo Moro’, Bari, Italy; am Dept. of Psychiatry, Psychosomatic Medicine and Psychotherapy, Goethe University Frankfurt, Frankfurt, Germany; an Department of Psychiatry, University of Basel, Basel, Switzerland; ao University of Stellenbosch, Cape Town, Western Province, South Africa; ap Seoul National University Hospital, Seoul, Republic of Korea; aq Yeongeon Student Support Center, Seoul National University College of Medicine, Seoul, Republic of Korea; ar Department of Psychiatry, Seoul National University College of Medicine, Seoul, Republic of Korea; as Department of Brain & Cognitive Sciences, College of Natural Sciences, Seoul National University, Seoul, Republic of Korea; at Department of Psychiatry, University of Münster, Münster, Germany; au Department of Child and Adolescent Psychiatry, Institute of Psychiatry and Mental Health, Hospital General Universitario Gregorio Marañón, Madrid, Spain; av Instituto de Investigación Sanitaria Gregorio Marañón (IiSGM), Madrid, Spain; aw School of Medicine, Universidad Complutense, Madrid, Spain; ax Georgia State University, Atlanta, GA, USA; ay Tri-institutional Center for Translational Research in Neuroimaging and Data Science (TReNDS), Georgia State, Georgia Tech, Emory, Atlanta, GA, USA; az Technische Universität Dresden, Faculty of Medicine, Division of Psychological and Social Medicine, Dresden, Germany; ba Departments of Psychiatry, Johns Hopkins School of Medicine, Baltimore, MD, USA; bb Department of Neuropsychiatry, University of Toyama Graduate School of Medicine and Pharmaceutical Sciences, Toyama, Japan; bc Department of Mental Health, Johns Hopkins Bloomberg School of Public Health, Baltimore, MD, USA; bd Departments of Psychiatry, Neuroscience, and Biomedical Engineering, Johns Hopkins School of Medicine, Baltimore, MD, USA; be School of Medical Sciences, University of New South Wales, Sydney, NSW, Australia; bf Mental Health Research Center, Moscow, Russia; bg Department of Psychiatry, Psychotherapy and Psychosomatics, Psychiatric Hospital, University of Zurich, Zurich, Switzerland; bh Montreal Neurological Institute, McGill University, Montreal, Canada; bi National Institute of Mental Health, Klecany, Czech Republic; bj Institute of Computer Science, Czech Academy of Sciences, Prague, Czech Republic; bk Faculty of Electrical Engineering, Czech Technical University in Prague, Prague, Czech Republic; bl MR Unit, Department of Diagnostic and Interventional Radiology, Institute for Clinical and Experimental Medicine, Prague, Czech Republic; bm Department of Psychiatry and Neuropsychology, Maastricht University, Maastricht, The Netherlands; bn Department of Psychiatry, University of Oxford, Oxford, UK; bo Department of Psychiatry and Human Behavior, University of California Irvine, Irvine, CA, USA; bp SAMRC Unit on Risk & Resilience in Mental Disorders, Dept of Psychiatry and Neuroscience Institute, University of Cape Town, Cape Town, Western Province, South Africa; bq Department of Psychiatry and Mental Health, University of Cape Town, Cape Town, Western Cape, South Africa; br Neuroscience Institute, University of Cape Town, Cape Town, Western Cape, South Africa; bs Department of Child and Adolescent Psychiatry, Technische Universität Dresden, Dresden, Germany; bt Valkenburg Hospital, Observatory, Cape Town, Western Cape, South Africa; bu Research Group in Psychiatry GIPSI, Department of Psychiatry, Faculty of Medicine, Universidad de Antioquia, Medellín, Antioquia, Colombia; bv Mood Disorders Program, Hospital Universitario San Vicente Fundación, Medellin, Colombia; bw Center of Neuroimmunology. Laboratory of Advanced Imaging in Neuroimmunological Diseases. Hospital Clinic de Barcelona, Barcelona, Spain; bx Institut d’Investigacions Biomèdiques August Pi i Sunyer (IDIBAPS), Barcelona, Spain; by Imaging Genetics Center, Mark & Mary Stevens Neuroimaging & Informatics Institute, Keck School of Medicine, University of Southern California, Los Angeles, CA, USA; bz Imaging Genetics Center, Department of Neurology, University of Southern California, Los Angeles, CA, USA; ca Clinical Translational Neuroscience Laboratory, Department of Psychiatry and Human Behavior, University of California Irvine, Irvine, CA, USA; cb Center for the Neurobiology of Learning and Memory, University of California Irvine, 309 Qureshey Research Lab, Irvine, CA, 92697, USA; cc Department of Psychiatry and Psychotherapy, University Lübeck, Germany; cd Department of Psychiatry and Clinical Psychology, Third Faculty of Medicine, Charles University, Prague, Czech Republic

**Keywords:** Brain, Cortical thickness, Gray matter, Mega-analysis, Neuroimaging, Schizophrenia, Volume

## Abstract

A common limitation of neuroimaging studies is their small sample sizes. To overcome this hurdle, the Enhancing Neuro Imaging Genetics through Meta-Analysis (ENIGMA) Consortium combines neuroimaging data from many institutions worldwide. However, this introduces heterogeneity due to different scanning devices and sequences. ENIGMA projects commonly address this heterogeneity with random-effects meta-analysis or mixed-effects mega-analysis. Here we tested whether the batch adjustment method, ComBat, can further reduce site-related heterogeneity and thus increase statistical power. We conducted random-effects meta-analyses, mixed-effects mega-analyses and ComBat mega-analyses to compare cortical thickness, surface area and subcortical volumes between 2897 individuals with a diagnosis of schizophrenia and 3141 healthy controls from 33 sites. Specifically, we compared the imaging data between individuals with schizophrenia and healthy controls, covarying for age and sex. The use of ComBat substantially increased the statistical significance of the findings as compared to random-effects meta-analyses. The findings were more similar when comparing ComBat with mixed-effects mega-analysis, although ComBat still slightly increased the statistical significance. ComBat also showed increased statistical power when we repeated the analyses with fewer sites. Results were nearly identical when we applied the ComBat harmonization separately for cortical thickness, cortical surface area and subcortical volumes. Therefore, we recommend applying the ComBat function to attenuate potential effects of site in ENIGMA projects and other multi-site structural imaging work. We provide easy-to-use functions in R that work even if imaging data are partially missing in some brain regions, and they can be trained with one data set and then applied to another (a requirement for some analyses such as machine learning).

## 1. Introduction

After the early reporting of ventricular enlargement in patients with schizophrenia (SCZ) using pneumoencephalography (Huber, 1957), there has been an exponential increase in the number of studies that use imaging techniques to detect brain differences in people with psychiatric disorders. This increase is most evident for studies using magnetic resonance imaging (MRI), probably due to its high resolution and its wide availability around the globe. However, most MRI studies have examined relatively small sample sizes, a limitation that may prevent the detection of true differences (type II errors), and because of the use of liberal thresholds, may even lead to increased detection of false differences (type I errors). Consequently, reports of unreliable, inconsistent and even contradictory results are not uncommon (Radua and Mataix-Cols, 2012).

Collaborative multi-site initiatives provide an opportunity to assemble larger and more diverse groups of subjects, leading to increased power and findings that may be more representative of the general population. Among these initiatives, the ENIGMA (Enhancing Neuro Imaging Genetics through Meta-Analysis; http://enigma.ini.usc.edu) Consortium (Thompson et al., 2014) stands out for including hundreds of groups worldwide and facilitating the sharing of tens of thousands of neuroimages. One great advantage of this consortium is the harmonization of the protocols to pre-process the MRI data, which has decreased the heterogeneity between the sites related to methodological factors. All sites apply the same pre-processing pipelines to obtain thickness and surface area estimates for cortical regions of interest (ROI) and volume estimates for subcortical ROIs; similar harmonized protocols are in use for standardized analysis of diffusion MRI, resting state fMRI and EEG data, as well as various kinds of omics data (GWAS and epigenetic data).

However, even though all sites participating in an ENIGMA project apply the same pre-processing protocol, data from different sites still show relevant methodological heterogeneity due to systematic differences in MRI scanning devices and acquisition sequences. Also, prior studies have reported that the results of the FreeSurfer segmentation process, for morphometric analysis of MRI, can be affected even by using different FreeSurfer versions, workstations or operating systems (Chepkoech et al., 2016; Gronenschild et al., 2012). Most ENIGMA projects address this residual heterogeneity by random-effects meta-analysis (RE-Meta), but estimation and control of heterogeneity in site-aggregated meta-analyses may be suboptimal (Chen and Benedetti, 2017). It is worth noting that a few ENIGMA studies have analyzed shared individual data (rather than site-aggregated statistical data). These “mega-analyses” of individual data considered the “site” as a random factor within a linear mixed-effects model (ME-Mega), and in several cases examined so far, showed higher statistical power than RE-Meta (Boedhoe et al., 2017, 2018; Favre et al., 2019; van Rooij et al., 2018) (Table 1).

Here, we tested whether ME-Mega may be further improved using a recently developed method to control for batch effects. Standard ME-Mega assumes that the error terms follow the same normal distribution at all sites, which is rarely the case as sites usually have different error variances. In addition, both RE-Mega and ME-Meta estimate the heterogeneity of each ROI independently, while it is likely that all ROIs share some heterogeneity. One method that overcomes these issues is ComBat (Johnson et al., 2007), a batch adjustment method developed for genomics data. Fortin and colleagues have shown that ComBat mega-analysis (ComBat-Mega) outperformed other methods for removing the effects of site from cortical thickness data obtained using the ANTs cortical thickness pipeline (Tustison et al., 2014) from a moderately small number of different sites (≤7 sites). Specifically, ComBat decreased scan-related heterogeneity and increased statistical power and reproducibility (Fortin et al., 2018). The current study examines whether this harmonization result can be extended to ENIGMA data obtained using a standardized FreeSurfer pipeline (Dale et al., 1999; Fischl et al., 1999). Moreover, we did not know whether the use of a larger number of sites could minimize the advantages of ComBat-Mega as compared to ME-Mega. To answer these questions, we analyzed the main structural MRI data from the ENIGMA Schizophrenia Working Group using RE-Meta, ME-Mega and ComBat-Mega, and then compared the findings. The RE-Meta of these data have been already published (van Erp et al., 2016, 2018; Wong et al., 2019); in those analyses, individuals with SCZ showed widespread thinner cortex and smaller surface area, as well as smaller hippocampus, amygdala, thalamus and accumbens volumes, and larger pallidum and lateral ventricle volumes.

We hypothesized that ComBat-Mega would show improvements over RE-Meta and ME-Mega in detecting differences between groups of individuals with SCZ and healthy controls (CON), with standard errors of these effects scaling by method: Combat-Mega < ME-Mega < RE-Meta. We further provide the R code (http://enigma.ini.usc.edu/wp-content/uploads/combat_for_ENIGMA_sMRI/combat_for_ENIGMA_sMRI.R) for the application of ComBat harmonization for other ENIGMA mega-analyses or other multi-site structural imaging work even if the imaging data are partially missing in some ROIs (the original ComBat function did not accept missing data).

## 2. Methods

### 2.1. Methodological approaches

Before detailing the collection of data and analyses in the present study, we will briefly explain the three methodological approaches. To exemplify the explanation, we will refer to a simple comparison of cortical thickness between groups of individuals with SCZ and CON, after covarying for effects of age and sex, but the concepts are applicable to other measures and statistical contrasts. We also conducted an alternative analysis covarying for age, sex, and intracranial volume (ICV).

#### 2.1.1. The RE-Meta approach

In the random-effects meta-analysis (RE-Meta), a linear model estimates the difference in cortical thickness between SCZ and CON for each ROI at each site, covarying for age and sex:

$$y_{r,i,j}=\alpha_{r,i}+X_{i,j}\cdot \beta_{r,i}+\varepsilon_{r,i,j}$$

where *y*_*r,i,j*_ is the measurement of cortical thickness of the *r*th ROI from the *j*th individual of the *i*th site, *α*_*r,i*_ is the estimate overall cortical thickness of the *r*th ROI from individuals of the *i*th site, *X*_*i,j*_ are the values of the variables (disorder, age, and sex) of the *j*th individual of the *i*th site, *β*_*r,i*_ are the estimates of the coefficients of these variables for the *r*th ROI from individuals of the *i*th site, and *ε*_*r,i,j*_ is the error term for the *r*th ROI in the *j*th individual of the *i*th site.

Estimates of coefficients of interest (e.g., *β*_*r*,*i*,1_, the difference between SCZ and CON are then pooled to obtain a single estimate for each ROI (*β*_*r*,*meta*,1_). A typical method to pool the coefficients is the weighted mean of the coefficient of each site (Radua and Mataix-Cols, 2012):

$$\beta_{r,\text{meta},1}=\sum_{i\in \text{sites}}\left(w_{r,i}\cdot \beta_{r,i,1}\right)$$

where *w*_*r,i*_ the weight of *i*th site for the *r*th ROI, and is calculated as the inverse of the variance of *β*_*r*,*i*,1_, plus the heterogeneity for the *r*th ROI ($\tau_{r}^{2}$):

$$w_{r,i}=\frac{1}{\text{var}\left(\beta_{r,i,1}\right)+\tau_{r}^{2}}$$

Frequently, the analyst does not use the coefficients but effect sizes, such as Hedges’ *g* (Radua and Mataix-Cols, 2012), but the concept is similar.

Some problems of RE-Meta are that *β*_*r,i*_ may be poorly estimated in sites with small sample sizes, or that $\tau_{r}^{2}$ may be poorly estimated in some scenarios (Chen and Benedetti, 2017).

#### 2.1.2. The ME-Mega approach

In the standard mixed effects mega-analysis (ME-Mega), a linear mixed-effects model is performed on shared individual subject data to estimate the overall difference in cortical thickness between SCZ and CON, for each ROI, covarying for age and sex. This analysis is conducted in a single step, with “site” included in the model as a random factor:

$$y_{r,i,j}=\alpha_{r}+X_{i,j}\cdot \beta_{r}+\gamma_{r,i}+\varepsilon_{r,i,j}$$

where *α*_*r*_ is the estimate overall cortical thickness of the *r*th ROI from all individuals, *β*_*r*_ are the estimates of the coefficients of the variables for the *r*th ROI from all individuals, and *γ*_*r,i*_ are the additive effects of the *i*th site in the *r*th ROI.

This approach benefits from a more robust estimation of *α*_*r*_ and *β*_*r*_ as it is based on the data from all sites, as well as from a more precise estimation of the heterogeneity. However, it still may have some minor issues. It assumes that the error terms follow the same normal distribution at all sites, which may seldom be the case. We acknowledge that it is possible to create linear mixed-effects models that consider a different variance for each site, but they involve the specification of variance structures for each statistical test, which may substantially complicate the analyses. In addition, the effects of site are estimated independently for each ROI, which may be suboptimal because the effects of site, even if different for each ROI, may still share some commonalities (e.g., an MRI device may yield a better signal contrast than another across the brain).

#### 2.1.3. The ComBat-mega approach

As compared to ME-Mega, the Combat mega-analysis (ComBat-Mega) assumes that the error terms may follow varying normal distributions at different sites:

$$y_{r,i,j}=\alpha_{r}+X_{i,j}\cdot \beta_{r}+\gamma_{r,i}+\delta_{r.i}+\varepsilon_{r,i,j}$$

where *δ*_*r,i*_ are the multiplicative effects of the *i*th site in the *r*th ROI.

In addition, it assumes that the additive and multiplicative effects of the sites are not completely independent across ROIs but, rather, they share a common distribution. Such considerations prevent the use of standard linear models, but ComBat uses an empirical Bayes framework to estimate the distribution of the effects of site (Johnson et al., 2007). Once estimated, it derives the additive error terms:

$$\varepsilon_{r,i,j}=\frac{y_{r,i,j}-\alpha_{r}-X_{i,j}\cdot \beta_{r}-\gamma_{r,i}}{\delta_{r.i}}$$

These terms allow the derivation of harmonized data:

$$y_{r,i,j}^{\text{ComBat}}=\alpha_{r}+X_{i,j}\cdot \beta_{r}+\varepsilon_{r,i,j}$$

These simpler data can then be analyzed with standard linear models to estimate the overall difference in cortical thickness between SCZ and CON groups, for each ROI.

### 2.2. Modifications of the ComBat function

Fortin and colleagues modified the original “combat” function, in the “sva” package for R (Leek et al., 2019), so that it could be applied to imaging data (Fortin et al., 2017). However, Fortin’s “combat” function may not be easily applicable to ENIGMA projects as it requires that the dataset has no missing data, which is seldom the case. In addition, it finds the harmonization parameters and applies them to the data within the same function, while some analyses - such as machine learning - require that the parameters are found in a training set and later applied to an independent test set (this is not the case here, but it might be the case in future studies). We further modified the “combat” function to allow for missing data and to separate the fitting and the application of the harmonization.

First, we divided the function into two subfunctions: “combat_fit”, which finds the harmonization parameters, and “combat_apply”, which applies them to the same or to another set. The “combat_fit” function automatically imputes missing data so that the function can find the harmonization parameters without errors. These imputations are predictions based on linear models of the ROI values by the covariates, separately for each ROI and each site:

$$y_{r,i,j}=\alpha_{r,i}+X_{i,j}\cdot \beta_{r,i}$$

The covariates are the variables introduced into the “combat_fit” function, which in the present study were the diagnosis, age, and sex. The “combat_fit” function also discards ROIs with no variance, which returned errors in the previous “combat” function. Importantly, these imputations are temporary and only aimed to avoid errors during the fitting of the parameters, they are not saved. To apply the parameters, the user must use the “combat_apply” function with the original data, and missing values are not imputed.

The reader may download the adapted ComBat functions for R from http://enigma.ini.usc.edu/wp-content/uploads/combat_for_ENIGMA_sMRI/combat_for_ENIGMA_sMRI.R.

### 2.3. Collection of data

The data for this paper includes the cortical thickness, surface area and subcortical volumes from 33 sites of the ENIGMA Schizophrenia Working Group (van Erp et al., 2016, 2018; Wong et al., 2019) who shared individual subject level FreeSurfer data for this project. The overall sample included 2897 individuals with a diagnosis of SCZ (mean age 34 years, 34% females) and 3141 CON (mean age 33 years, 49% females). For SCZ, the mean age of onset was 23 years and their Positive and Negative Syndrome Scale (PANSS) (Kay et al., 1987) scores for total/positive/negative symptoms were 61/16/17, respectively. The researchers at each of the sites had collected the data after obtaining participants’ written informed consent, with protocols that had been approved by local institutional review boards. We provide a description of the overall sample in Table 2 and a description of the sample from each site in Supplementary Table S1.

All sites had processed the data with FreeSurfer (Fischl, 2012) versions 4.0 to 5.3, except for version 5.2 which was found to produce low intra-class correlations compared to the other versions, and within site all patients and controls were processed using the same FreeSurfer version (van Erp et al., 2016, 2018) according to the ENIGMA protocols, which are available at http://enigma.usc.edu/protocols/imaging-protocols. For cortical ROIs, they involved the estimation of cortical vertex-wise statistics, the extraction of cortical thickness and surface area for 70 Desikan-Killiany (DK) atlas regions (Desikan et al., 2006), and quality checks (van Erp et al., 2018). For subcortical ROIs, they involved the estimation of subcortical volumes and quality checks (van Erp et al., 2016).

### 2.4. Statistical analyses

We conducted comparisons of MRI data between individuals with SCZ and CON to assess the statistical significance, power and familywise error rate (FWER) using RE-Meta, ME-Mega and ComBat-Mega. We formally tested whether ComBat-Mega increases the statistical significance and power of the differences between individuals with SCZ and CON by attenuating site-effects, using a permutation test and a small-subset strategy respectively. We also used the data of the permutation test to check the FWER.

#### 2.4.1. Comparisons of MRI data between individuals with SCZ and CON

We conducted the RE-Meta in two steps. In the first step, we compared the values of each ROI between SCZ and CON via a standard linear model, with age and sex as covariates, separately for each site. We then converted the difference to a Hedges’ *g* and its variance for each site and ROI. In the second step, we conducted a random-effects meta-analysis of the Hedges’ *g* of each ROI with the “metafor” package for R (Viechtbauer, 2010), and we corrected the *p*-values for multiple comparisons with the Holm method.

For ME-Mega, we compared the values of each ROI between SCZ and CON via a linear mixed-effects model, with age and sex as covariates and site as a random factor, with the “lme4” and “lmerTest” packages for R (Bates et al., 2015; Kuznetsova et al., 2017). We then divided the difference by the standard deviation (derived from the model) and corrected it for small-sample bias to obtain a Hedges’ *g* and its variance, and we corrected the *p*-values for multiple comparisons using the Holm method (Holm, 1979).

Finally, for ComBat-Mega, we first removed the effects of site using the ComBat functions (modelling the effects of diagnosis, age, and sex), and then compared the values of each ROI (e.g., cortical thickness of the frontal pole) between SCZ and CON via a standard linear model, with age and sex as covariates. Note that the ComBat functions use covariates (e.g., age and sex) to better estimate the effects of site, but they do not remove the effects of these covariates; for this reason, we included these covariates in the subsequent linear model. As for ME-Mega, we converted the difference to a Hedges’ *g* and its variance, and we corrected the *p*-values for multiple comparisons with the Holm method. Note that we applied a single ComBat harmonization for different types of data (cortical thickness, cortical surface area, and subcortical volume) because we considered that they were related. We also conducted an alternative analysis with a separate harmonization for each type of data.

#### 2.4.2. Comparison of the statistical significance

To test whether ComBat-Mega had improved the statistical significance we used a permutation approach. We followed the Draper-Stoneman procedure, which according to results from a study comparing different algorithms (Winkler et al., 2014), is one of the procedures that best controls the FWER and that can be safely applied here. Note that other algorithms such as Freedman Lane would produce different permuted data for RE-Meta, ME-Mega and ComBat-Mega, which would be problematic in our study because these unwanted differences could confound other potential differences between the methods. Specifically, we randomly permuted the diagnosis among the individuals within each site and repeated all comparison analysis 1000 times.

**Fig. 1.**
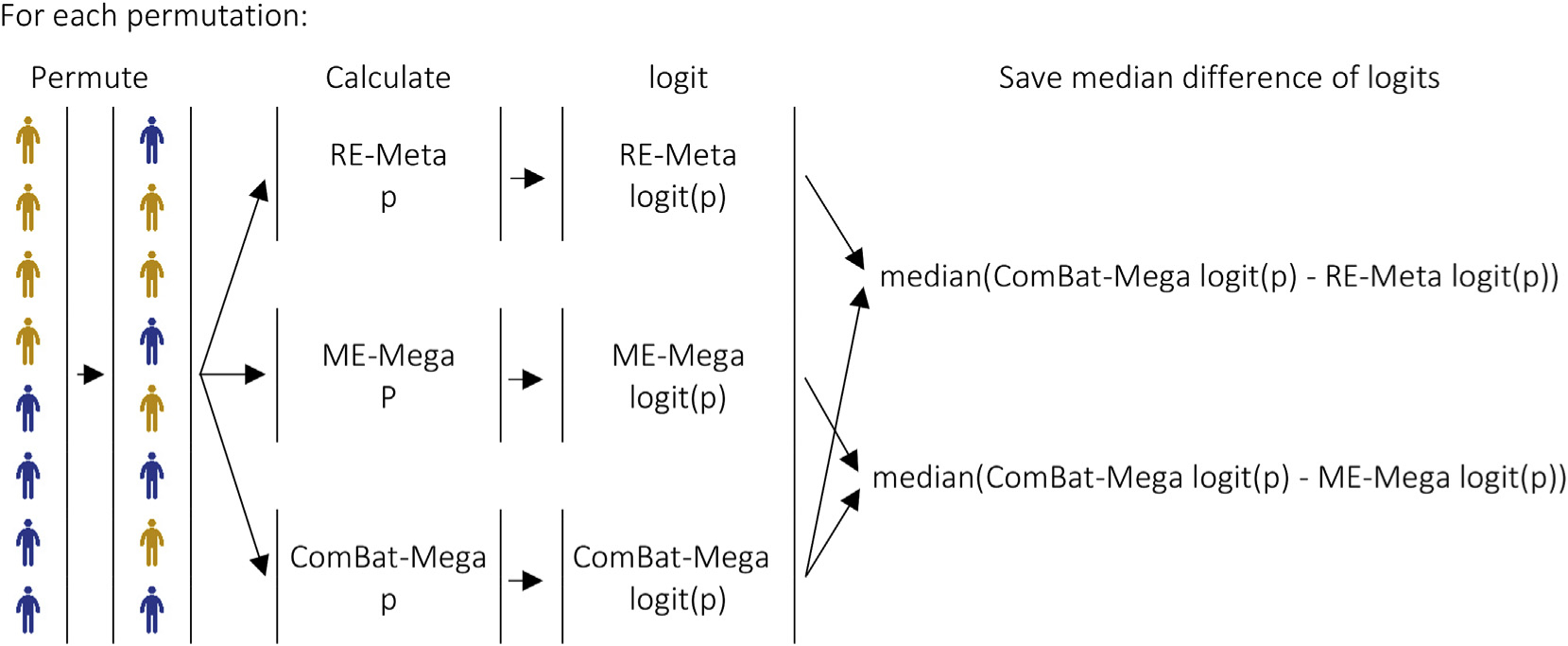
Steps of each iteration of the permutation test used to compare the statistical significance between random-effects meta-analysis, mixed-effects mega-analysis and ComBat mega-analysis *Footnote:* ComBat-Mega: ComBat mega-analysis; ME-Mega: mixed-effects mega-analysis; RE-Meta: random-effects meta-analysis.

To show the differences in statistical significance between methods expected by chance, we plotted the histogram of the median difference in the logit-transformed *p*-values between the methods across the permutations (Fig. 1). For example, in one permutation we randomly assigned study participants to patient or control status. We then compared these randomly assigned patients and controls using RE-Meta, ME-Mega and ComBat-Mega. We then calculated differences between logit-transformed p-values of the ComBat-Mega comparison and logit-transformed p-values of the RE-Meta (or ME-Mega) comparisons for each ROI. From these, we only saved the median between logit-transformed p-value difference. Note that this median difference should be very close to zero, given that participant assignment was random, and there should therefore be no patient-control group differences other than by chance. By conducting multiple of these permutations, we were able plot the histogram of the median differences expected by chance alone. Finally, we compared the median difference of the original analysis (with correctly assigned patient and control status) with the histogram of the median differences expected by chance. Only median differences were used in this analysis to simplify the test as doing so avoids the need to correct for multiple comparisons.

We must note that without the logit (or other) transforms, the detection of differences in statistical significance would be too sensitive for large *p*-values and too little sensitive for small *p*-values. For example, if the (non-transformed) *p*-value using one approach was 0.6 and the (non-transformed) *p*-value using another approach was 0.4, the difference in *p*-values would be very large (0.6–0.4 = 0.2) even if the two *p*-values might be considered conceptually very similar, whereas if the (non-transformed) *p*-value using one approach was 0.003 and the (non-transformed) *p*-value using another approach was 0.001, the difference in *p*-values would be very small (0.003–0.001 = 0.002) even if one *p*-value is three times the size of the other. With the logit transform, the *p*-values of the first example would be 0.4 and −0.4, with a difference of 0.8, and the *p*-values of the second example would be −5.8 and −6.9, with a difference of 1.1.

The use of a permutation test implied that both the estimated probability of obtaining the observed median difference in (logit-transform) *p*-values was discrete, i.e., it could only be 0.001, or 0.002, or 0.003, etcetera. However, we were only interested in assessing if this estimation was <0.05, for what this level of precision should not pose any problems.

#### 2.4.3. Evaluation of the statistical power

We also tested whether ComBat-Mega increases the statistical power using a small-subset strategy. Specifically, we repeated 500 times the analyses but including each time only a random sample of 10 sites. We then counted the number of times that these analyses using only 10 sites were able to detect differences between SCZ and CON. We only used ROIs in which the differences between SCZ and CON were strongly statistically significant in the main analyses using the 33 sites (FWER<0.001 for RE-Meta, for ME-Mega, and for ComBat-Mega), as we assumed that they have true differences. Finally, we conducted a Wilcoxon signed-ranked test to compare the statistical power across ROIs between ComBat-Mega and RE-Meta, as well as between ComBat-Mega and ME-Mega.

#### 2.4.4. Determination of the empirical FWER

We also used the permutation data created above to check whether the FWER for the three methods were appropriate, i.e., we counted the proportion of permutations in which at least one ROI had a Holm-corrected *p*-value < 0.05. Again, the use of a permutation test implied that the estimated FWER was discrete, but we were only interested in assessing whether it was <0.05.

## 3. Results

With ComBat-Mega, on average, individuals with a diagnosis of SCZ showed thinner cortex and smaller surface area in nearly all cortical ROIs (Table 3). The only exceptions were the bilateral pericalcarine fissures and right entorhinal cortex (where between-group differences in thickness did not reach statistical significance after correction for multiple comparisons) and the left isthmus of the cingulate and right temporal pole (where between-group differences in surface area did not reach statistical significance after correction for multiple comparisons). The SCZ group also showed, on average, smaller bilateral thalamus, hippocampus, amygdala, and right accumbens volumes, and larger bilateral lateral ventricle, putamen, and pallidum volumes. Smaller left accumbens and larger bilateral caudate volumes were not statistically significant after correction for multiple comparisons.

Results were in the same direction for the RE-Meta and ME-Mega, though RE-Meta did not detect thinner cortex in three ROIs (bilateral rostral anterior cingulate and left caudal anterior cingulate) and smaller surface area in six ROIs (bilateral pericalcarine fissure, left posterior cingulate and temporal pole, and right isthmus cingulate and insula).

**Fig. 2.**
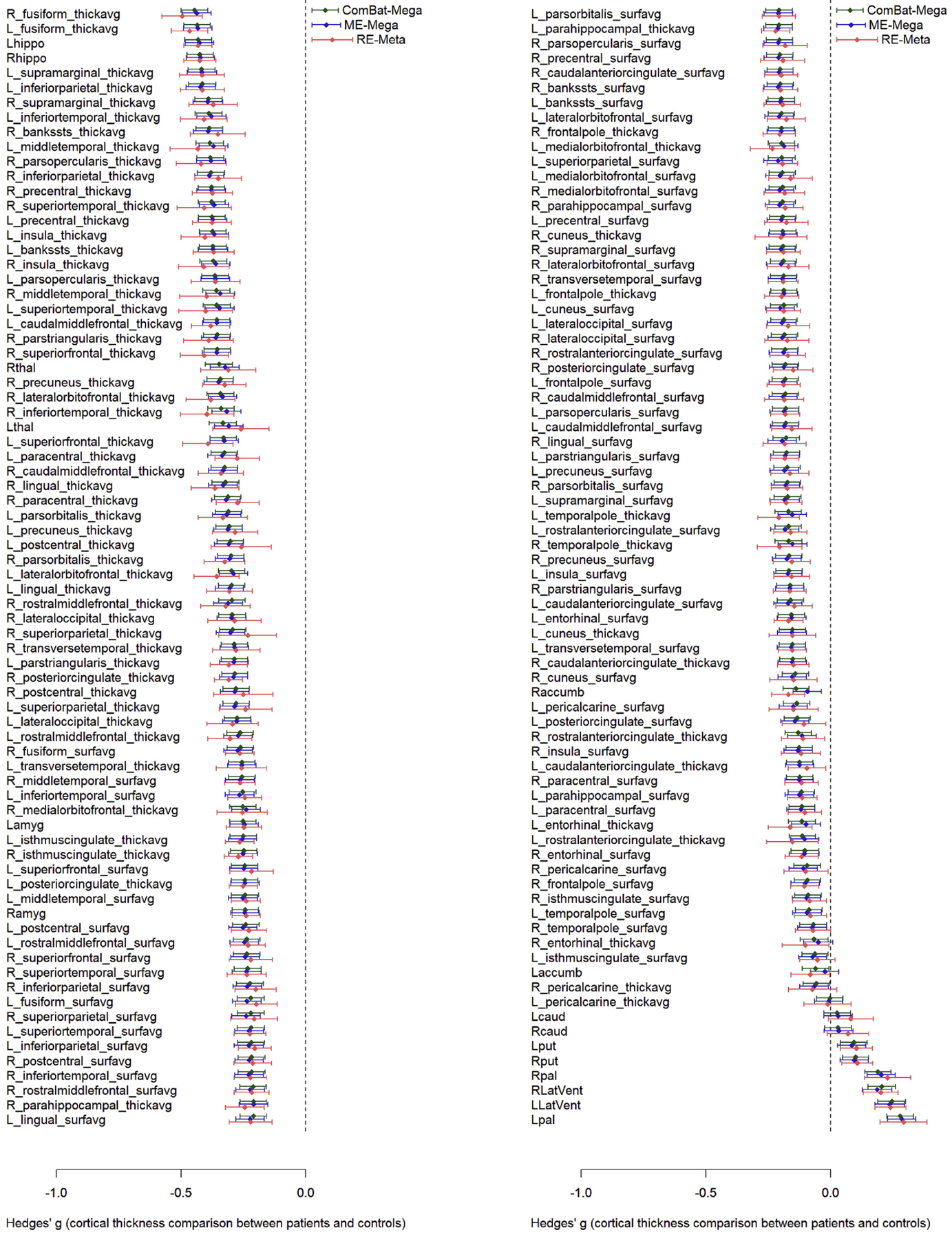
Forest plot for random-effect meta-analysis (light red), mixed-effects mega-analysis (blue) and ComBat mega-analysis (dark green). *Footnote:* The width of the confidence intervals in the legend corresponds to the mean width of the confidence intervals across the brain. ComBat-Mega: ComBat mega-analysis; ME-Mega: mixed-effects mega-analysis; RE-Meta: random-effects meta-analysis.

**Fig. 3.**
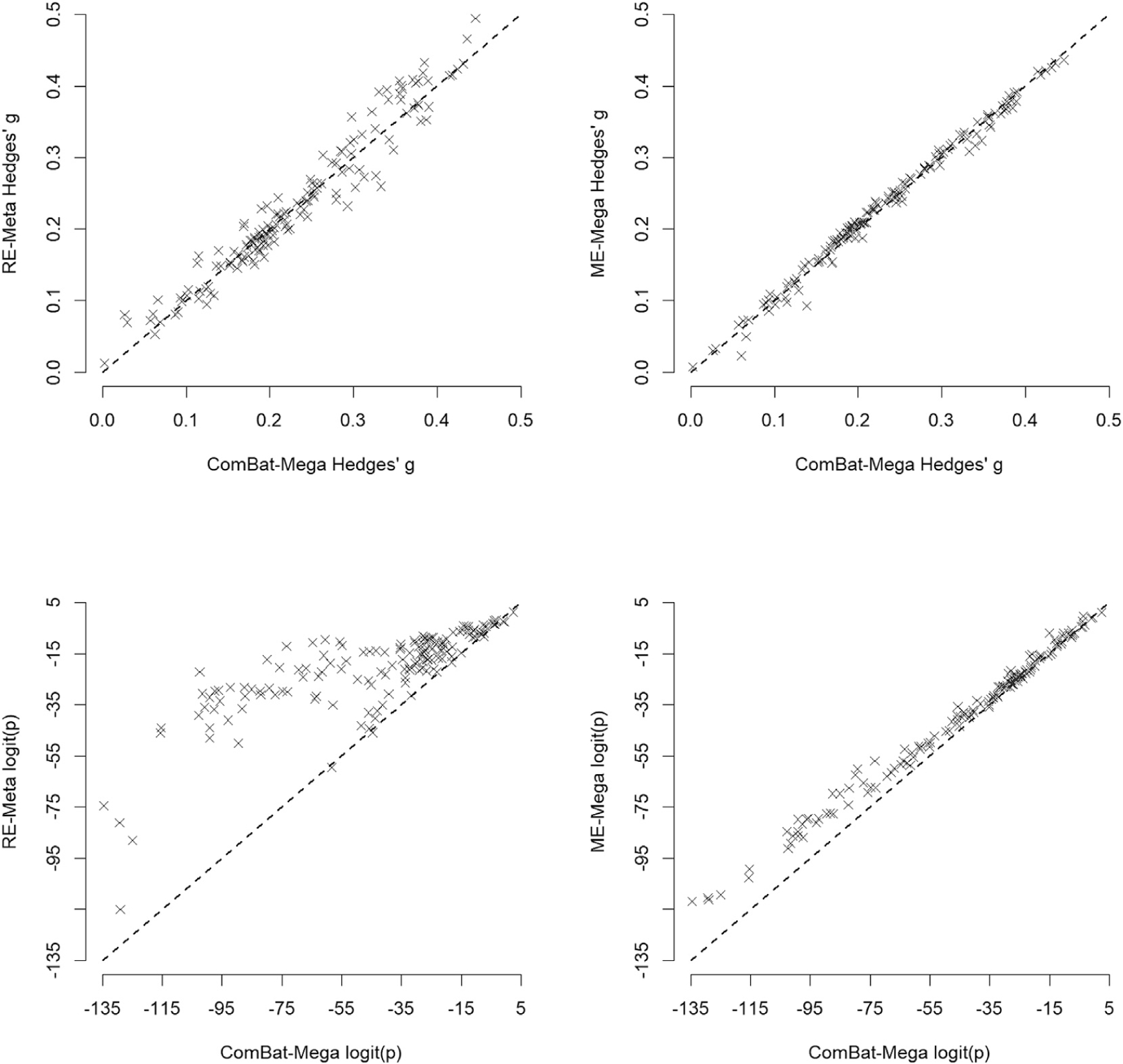
Hedges’ *g* and *p*-values of random-effect meta-analysis, mixed-effects mega-analysis and ComBat mega-analysis in the comparison of ENIGMA brain data between 2897 patients with schizophrenia and 3141 healthy controls. *Footnote:* Each cross represents an ROI. ComBat-Mega: ComBat mega-analysis; ME-Mega: mixed-effects mega-analysis; RE-Meta: random-effects meta-analysis. The top plots show that ComBat-Mega effect sizes are similar to RE-Meta and ME-Mega effect sizes, as crosses are mostly distributed around the diagonal lines. The bottom plots show that ComBat-Mega *p*-values are substantially smaller than RE-Meta *p*-values (crosses are clearly above the diagonal line), and slightly smaller than ME-Mega *p*-values (crosses tend to be slightly above the diagonal line).

The Hedges’ *g* estimates for the differences were similar across the different analytic methods, but their statistical significance was greater in ComBat-Mega as compared to RE-Meta and ME-Mega (Figs. 2 and 3). The difference in statistical significance was relatively minor when comparing ComBat-Mega to ME-Mega, whereas particularly relevant when comparing ComBat-Mega to RE-Meta (Fig. 3).

**Fig. 4.**
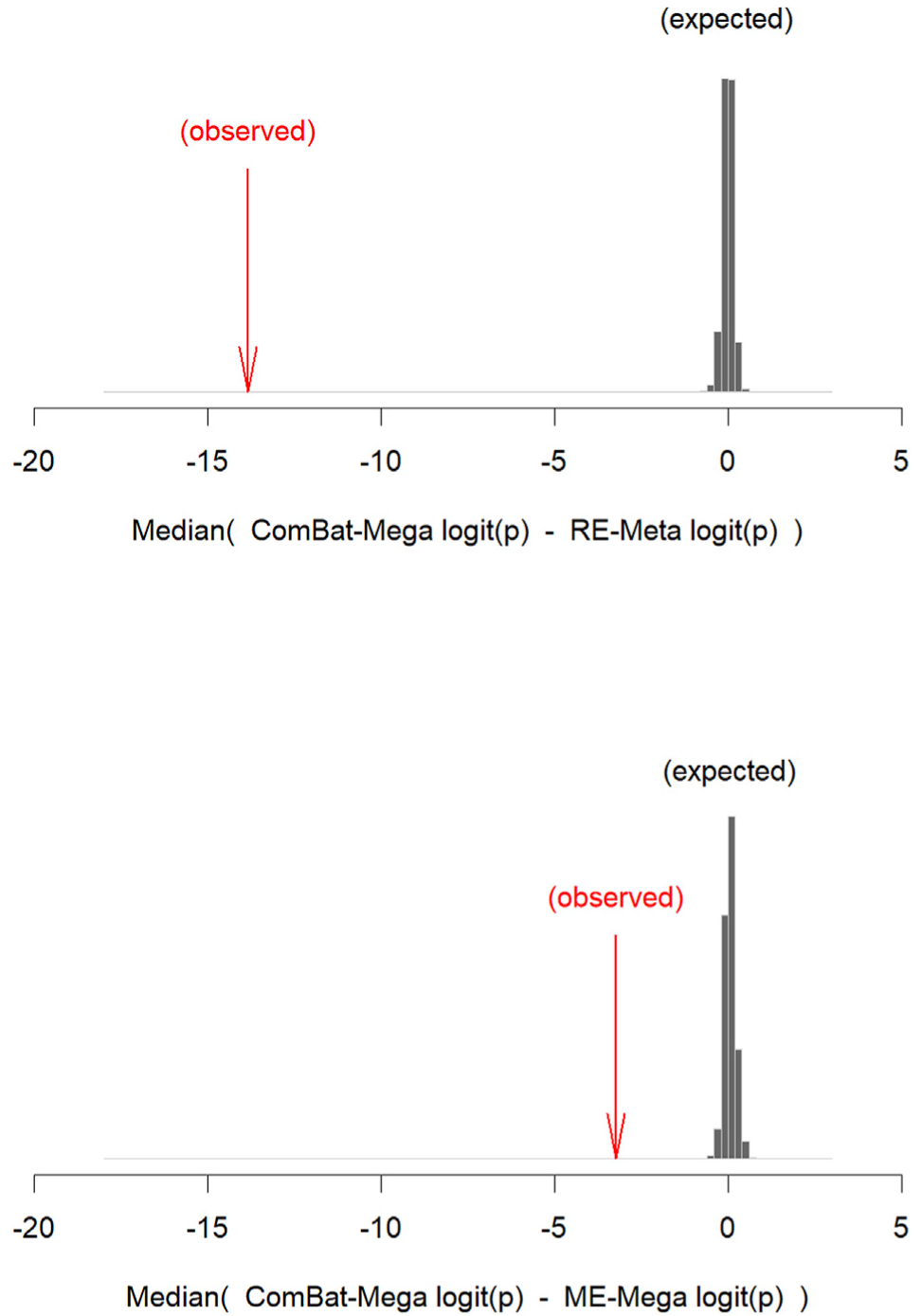
Median difference between logit-transformed *p*-values derived from ComBat mega-analysis and logit-transformed *p*-values derived from random-effects meta-analysis and mixed-effects mega-analysis in the original data (red) and in the permuted data (histograms). *Footnote:* ComBat-Mega: ComBat mega-analysis; ME-Mega: mixed-effects mega-analysis; RE-Meta: random-effects meta-analysis. The histograms (in gray) show the expected ComBat-Mega-related increase of statistical significance by chance, and the arrows (in red) show the actual increase. The latter is clearly larger than that former (negative values mean that ComBat-Mega increases statistical significance).

The median difference between logit-transformed ComBat-Mega *p*-values and logit-transformed RE-Meta *p*-values in the original data was 13.9. This was substantially larger than any of the median differences in the permuted data (all < 0.61), indicating that the higher statistical significance of ComBat-Mega findings was unlikely due to chance (probability 0.001) (Fig. 4). For the comparison between ComBat-Mega and ME-Mega, the median difference was smaller (3.2), but still unlikely due to chance (all median differences in the permuted data <0.52, probability 0.001).

**Fig. 5.**
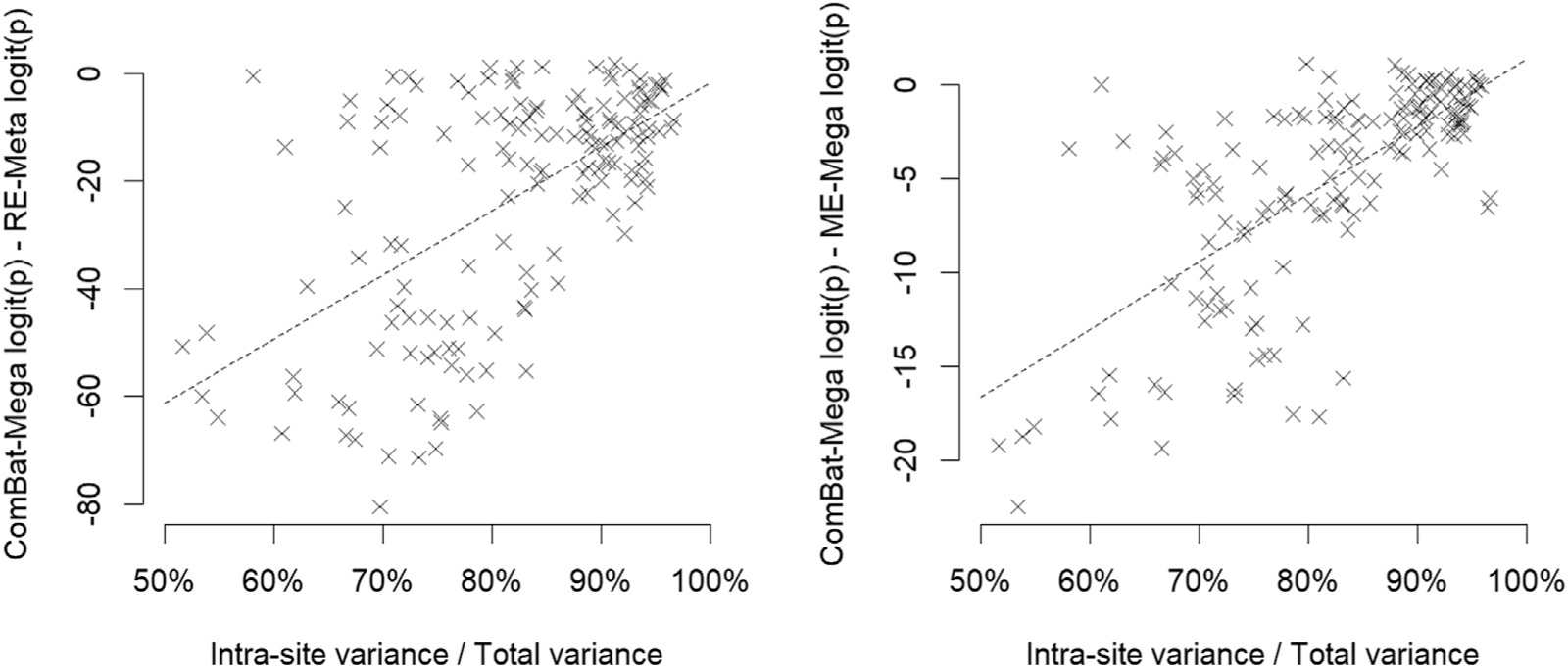
Relationship between the intra-site variance/total variance ratio and ComBat mega-analysis-related increase of statistical significance. *Footnote:* ComBat-Mega: ComBat mega-analysis; ME-Mega: mixed-effects mega-analysis; RE-Meta: random-effects meta-analysis. The ComBat-Mega-related increase of statistical significance (negative values in the Y axis) is larger in regions with lower intra-site variance/variance ratio (around 50–70%).

Interestingly, a plot of the ComBat-Mega-related increase in statistical significance as a function of the intra-site variance/total variance ratio, showed that the increase in statistical significance was larger in those ROIs in which intra-site variance was only ~50–70% of total variance compared to those ROIs in which intra-site variance was ~90–100% of total variance (p < 0.001, Fig. 5).

In the evaluation of statistical power using the small-subset strategy, the statistical power was higher for ComBat-Mega (statistical power = 83.5%) than for RE-Meta (statistical power = 53.7%; Wilcoxon *p*-value < 0.001) or ME-Mega (statistical power = 80.4%; Wilcoxon *p*-value < 0.001).

The empirical FWER was ≤0.05 for all analytic methods (RE-Meta: 0.024; ME-Mega: 0.027; ComBat-Mega: 0.025).

When we applied the ComBat harmonization separately for cortical thickness data, cortical surface area data and subcortical volume data, we found the same differences with nearly identical Hedges’ *g* (Supplementary Figure S1). The statistical significance was minimally lower (median difference between single ComBat logit-transformed *p*-values and separate ComBat logit-transformed *p*-values was 0.1), the statistical power in the small-subset strategy was 83.5%, and the empirical FWER was 0.026.

When we covaried ComBat-Mega by age, sex and ICV, results were similar: The only differences were that the right frontal pole, isthmus of the cingulate and pericalcarine and left parahippocampal and temporal pole decreases in surface area were no longer statistically significant, whereas the left pericalcarine decrease in surface area and the bilateral caudate increases in volume reached statistical significance. Results were again in the same direction for the RE-Meta and ME-Mega, though RE-Meta did not detect statistically significant differences in 36 of the ROIs showing differences with ComBat-Mega, and ME-Mega did not detect smaller right accumbens volume (and detected smaller surface area in left parahippocampal and right pericalcarine but not in left paracentral and right entorhinal). The Hedges’ *g* estimates for the differences were again similar across the different analytic methods, but their statistical significance was again greater in ComBat-Mega as compared to RE-Meta and ME-Mega (Supplementary Figure S2).

## 4. Discussion

In this study, we analyzed individual subject level data pooled by the ENIGMA Schizophrenia Working Group using three methods to account for the effects of site: random-effects meta-analysis (RE-Meta), linear mixed-effects models (ME-Mega), and ComBat harmonization followed by standard linear models (ComBat-Mega). The results of the comparison between SCZ and CON using ComBat-Mega were similar to the studies already published by the ENIGMA Schizophrenia Working Group: SCZ showed a widespread thinner cortex and smaller surface area (van Erp et al., 2018), smaller hippocampus, amygdala, thalamus and accumbens, and larger lateral ventricles, putamen and pallidum (van Erp et al., 2016) than CON. The results of the same comparison using RE-Meta and ME-Mega were in the same direction and had similar effect sizes, although with a lower statistical significance (i.e. wider confidence intervals, larger *p*-values), especially for RE-Meta. In other words, the use of ComBat increased the statistical significance (i.e., narrower confidence intervals, smaller *p*-values) of the differences between SCZ and CON. This was specially apparent in those ROIs in which intra-site variance was only ~50–70% of total variance. ComBat Mega also showed increased statistical power when we repeated the analyses with fewer sites. All approaches controlled well the FWER, even too strictly probably due to the use of the Holm method, which is more powerful than the Bonferroni method but still conservative (Blakesley et al., 2009). Findings were similar when covarying by ICV.

Based on these findings, we recommend that ENIGMA mega-analysis projects consider applying the ComBat function to reduce the effects of site, followed by standard statistical analysis without including site as a fixed or random effect in the statistical model. To apply ComBat harmonization, we provide easy-to-use functions for R that work even if there are missing data and they can be trained with data from one set and then applied to data from another.

We must note that we conducted these analyses with the three main types of data used in ENIGMA projects: thickness of cortical ROIs, surface area of cortical ROIs, and volumes of subcortical nuclei. However, some ENIGMA projects use other types of data, such as mean fractional anisotropy of white matter tracts, and we have not explored whether the application of ComBat would be beneficial for these projects. Two notions suggest that ComBat should be broadly beneficial. On the one hand, the ComBat algorithm is not specific for a given type of imaging data. Indeed, while it was developed for genomics data (Johnson et al., 2007), we here successfully applied it to three types of ENIGMA imaging data. Moreover, Fortin and colleagues found that ComBat outperforms other harmonization methods for voxel-based fractional anisotropy and mean diffusivity (Fortin et al., 2017), and Yu et al. found similar results for resting-state functional connectivity and network measures (Yu et al., 2018).

While our findings suggest that ComBat harmonization will be useful for most ENIGMA mega-analyses and other multi-site structural imaging work, we suggest caution when combining different types of data. We conducted a single ComBat harmonization for different types of MRI data because we considered that thickness, area, and volume are related, as they are obtained from the same FreeSurfer output of the T1-weighted image and all measure amounts of gray matter. Indeed, an alternative analysis with separate ComBat harmonization for each type of data yielded nearly identical results. However, we do not know whether the application of a single ComBat harmonization on other combinations of data would behave similarly.

Other popular approaches for pooling neuroimaging data are the voxel-based meta-analytic methods, such as Seed-based *d* Mapping (SDM) (Albajes-Eizagirre et al., 2019; Radua et al., 2012) or Activation Likelihood Estimation (ALE) (Eickhoff et al., 2009, 2012). These methods can include imaging studies even if they only report the coordinates of the peaks of the clusters of statistical significance. Therefore, a great advantage of these methods is the exhaustive inclusion of studies. In addition, the analyses are conducted at the voxel level (rather than using ROIs). These methods traditionally tested whether the reported findings tended to converge in a few brain voxels (Albaje-s-Eizagirre and Radua, 2018), but novel methods are able to directly test whether there are differences - even if they are widespread and do not converge (Albaje- s-Eizagirre et al., 2019). In view of the results of the present study, one could wonder whether these voxel-based methods should also conduct ComBat mega-analysis instead of meta-analysis. However, to use ComBat they would need access to individual subject level data, which at present are often not available. Another aspect to consider is whether we need SDM or ALE meta-analyses after an ENIGMA ComBat mega-analysis is published. Here, we must remember that SDM and ALE are voxel-based and include virtually all published studies, whereas most ENIGMA studies are ROI-based and include only the data that authors agree to share. Therefore, these different approaches present interesting complementary information.

Our study has some limitations. First, we already stated that we have not explored whether the application of ComBat would be beneficial for projects using other types of data, although several facts suggest that ComBat should be broadly beneficial. Second, we also acknowledged that we do not know whether the application of a single ComBat harmonization on other combinations of data would behave similarly. Third, our analysis is focused on the differences between SCZ and CON, whose distribution is roughly similar across sites. The effects of site and thus the importance of their removal might be larger for conditions with few cases in each site, where pooling data is more beneficial. Fourth, ComBat-Mega addresses some issues but not others, which still need to be investigated, such as site by nuisance confounds. For example, a site with poor quality data may also be a site with a mean age higher than other sites. Future studies addressing these questions could point to methods other than ComBat. Finally, there is a conceptual difference in the effects of site that are modeled in ComBat/ME-Mega and the effects of site that are modeled in RE-Meta. The former effects are in (individual) raw data and refer to site-specific constants that are added to or that multiply the measurements. The latter effects, conversely, are in (group) effect sizes, and are probably a mix of several factors such as site-specific constants that multiply the measurements, heterogeneity in the differences between SCZ and CON, or differences in precision between studies.

To conclude, this paper provides evidence of the superiority of ComBat harmonization over standard mega-analyses and meta-analyses in reducing site-related heterogeneity and thus increase statistical power. We therefore recommend that ENIGMA mega-analysis projects and other multi-site structural imaging work consider applying the ComBat function, which we provide employing easy functions for R. The provided code works with missing data and allows for harmonization of a test set based on the training set (a requirement for machine learning and possibly replication studies). We hope that future ENIGMA mega-analysis projects will improve between-site harmonization using ComBat.

## Supplementary Material

1

## Figures and Tables

**Table 1:** Previous ENIGMA projects that included both mega-analyses and meta-analyses.

	RE-Meta	ME-Mega
Subcortical volumes in obsessive-compulsive disorder (Boedhoe et al., 2017)	↓ in1 ROI and t in ↑ ROI	↓ in 1 ROI and ↑ in 1 ROI
Fractional anisotropy in bipolar disorder (Favre et al., 2019)	↓ in 23 out of 44 ROIs	↓ in 29 out of 44 ROIs
Cortical thickness in obsessive-compulsive disorder (Boedhoe et al., 2018)	No findings	↓ in 2 ROIs
Surface area in obsessive-compulsive disorder (Boedhoe et al., 2018)	↓ in 1 ROI	↓ in 1 ROI
Subcortical volumes in autism spectrum disorder (van Rooij et al., 2018)	↓ in 3 ROIs	↓ in 4 ROIs
Cortical thickness in autism spectrum disorder (van Rooij et al., 2018)	↑ in 3 ROIs ↓I in 10 ROIs	↑ in 9 ROIs and ↓ in 7 ROIs

*Footnote:* ROI: region of interest. ME-Mega: mixed-effects mega-analysis; RE-Meta: random-effects meta-analysis.

**Table 2:** Description of the overall sample.

	Sample size	Age (SD)	Females	Age of onset (SD)	Duration of illness (SD)	PANSS			SAPS (SD)	SANS (SD)	CDE (SD)
						Total (SD)	Positive (SD)	Negative (SD)			
Patients with schizophrenia	2897	33.9 (12.0)	34.2%	22.8 (7.1)	12.1 (12.5)	60.5 (25.3)	15.5 (6.8)	16.6 (7.8)	20.2 (18.5)	23.0 (16.9)	426 (591)
Healthy controls	3141	33.3 (13.2)	49.0%								

*Footnote:* CDE: chlorpromazine dose equivalent; PANSS: Positive and Negative Syndrome Scale; SANS: Scale for the Assessment of Negative Symptoms; SAPS: Scale for the Assessment of Positive Symptoms; SD: standard deviation.

**Table 3:** Effect sizes and confidence intervals derived from the ComBat mega-analysis.

		Thickness	Surface area
Bankssts	L	−0.37 (−0.43,−0.32)	−0.2 (−0.25,−0.14)
	R	−0.39 (−0.44,−0.33)	−0.2 (−0.26,−0.15)
Caudal anterior cingulate	L	−0.12 (−0.18,−0.07)	−0.16 (−0.21,−0.11)
	R	−0.15 (−0.2,−0.1)	−0.2 (−0.26,−0.15)
Caudal middle frontal	L	−0.36 (−0.41,−0.3)	−0.18 (−0.23,−0.13)
	R	−0.33 (−0.38,−0.27)	−0.18 (−0.23,−0.13)
Cuneus	L	−0.15 (−0.21,−0.1)	−0.19 (−0.24,−0.13)
	R	−0.19 (−0.24,−0.14)	−0.14 (−0.19,−0.09)
Entorhinal	L	−0.11 (−0.17,−0.06)	−0.16 (−0.21,−0.1)
	R	−0.07 (−0.12,−0.01)	−0.1 (−0.16,−0.05)
Frontal pole	L	−0.19 (−0.24,−0.13)	−0.18 (−0.23,−0.13)
	R	−0.2 (−0.25,−0.14)	−0.09 (−0.15,−0.04)
Fusiform	L	−0.44 (−0.49,−0.38)	−0.22 (−0.27,−0.17)
	R	−0.45 (−0.5,−0.39)	−0.26 (−0.32,−0.21)
Inferior parietal	L	−0.41 (−0.47,−0.36)	−0.22 (−0.27,−0.16)
	R	−0.38 (−0.43,−0.33)	−0.22 (−0.28,−0.17)
Inferior temporal	L	−0.39 (−0.44,−0.33)	−0.25 (−0.31,−0.2)
	R	−0.34 (−0.39,−0.29)	−0.22 (−0.27,−0.16)
Insula	L	−0.37 (−0.43,−0.32)	−0.17 (−0.22,−0.11)
	R	−0.37 (−0.42,−0.32)	−0.13 (−0.18,−0.07)
Isthmus cingulate	L	−0.25 (−0.3,−0.2)	−0.06 (−0.12,−0.01)
	R	−0.25 (−0.3,−0.2)	−0.09 (−0.14,−0.04)
Lateral occipital	L	−0.27 (−0.33,−0.22)	−0.19 (−0.24,−0.13)
	R	−0.29 (−0.35,−0.24)	−0.18 (−0.24,−0.13)
Lateral orbitofrontal	L	−0.3 (−0.35,−0.24)	−0.2 (−0.25,−0.14)
	R	−0.34 (−0.39,−0.29)	−0.19 (−0.24,−0.14)
Lingual	L	−0.3 (−0.35,−0.24)	−0.21 (−0.26,−0.16)
	R	−0.32 (−0.37,−0.27)	−0.18 (−0.23,−0.13)
Medial orbitofrontal	L	−0.2 (−0.25,−0.14)	−0.19 (−0.25,−0.14)
	R	−0.25 (−0.31,−0.2)	−0.19 (−0.25,−0.14)
Middle temporal	L	−0.38 (−0.44,−0.33)	−0.24 (−0.3,−0.19)
	R	−0.36 (−0.41,−0.3)	−0.26 (−0.31,−0.2)
Paracentral	L	−0.33 (−0.38,−0.27)	−0.11 (−0.17,−0.06)
	R	−0.31 (−0.37,−0.26)	−0.12 (−0.18,−0.07)
Parahippocampal	L	−0.21 (−0.26,−0.15)	−0.12 (−0.17,−0.06)
	R	−0.21 (−0.26,−0.16)	−0.19 (−0.25,−0.14)
Pars opercularis	L	−0.36 (−0.42,−0.31)	−0.18 (−0.23,−0.13)
	R	−0.38 (−0.44,−0.33)	−0.2 (−0.26,−0.15)
Pars orbitalis	L	−0.31 (−0.36,−0.26)	−0.21 (−0.26,−0.15)
	R	−0.3 (−0.35,−0.25)	−0.17 (−0.23,−0.12)
Pars triangularis	L	−0.29 (−0.34,−0.23)	−0.18 (−0.23,−0.12)
	R	−0.36 (−0.41,−0.3)	−0.16 (−0.22,−0.11)
Pericalcarine	L	0 (−0.06,0.05)	−0.14 (−0.19,−0.08)
	R	−0.06 (−0.11,0)	−0.09 (−0.15,−0.04)
Postcentral	L	−0.3 (−0.36,−0.25)	−0.24 (−0.29,−0.19)
	R	−0.28 (−0.33,−0.23)	−0.22 (−0.27,−0.16)
Posterior cingulate	L	−0.24 (−0.3,−0.19)	−0.13 (−0.19,−0.08)
	R	−0.28 (−0.34,−0.23)	−0.18 (−0.23,−0.13)
Precentral	L	−0.38 (−0.43,−0.32)	−0.19 (−0.24,−0.14)
	R	−0.38 (−0.43,−0.32)	−0.2 (−0.26,−0.15)
Precuneus	L	−0.31 (−0.36,−0.25)	−0.17 (−0.23,−0.12)
	R	−0.34 (−0.4,−0.29)	−0.17 (−0.22,−0.11)
Rostral anterior cingulate	L	−0.11 (−0.17,−0.06)	−0.17 (−0.22,−0.12)
	R	−0.13 (−0.18,−0.08)	−0.18 (−0.24,−0.13)
Rostral middle frontal	L	−0.26 (−0.32,−0.21)	−0.24 (−0.29,−0.18)
	R	−0.3 (−0.35,−0.24)	−0.21 (−0.26,−0.16)
Superior frontal	L	−0.33 (−0.38,−0.28)	−0.24 (−0.3,−0.19)
	R	−0.35 (−0.41,−0.3)	−0.24 (−0.29,−0.18)
Superior parietal	L	−0.28 (−0.33,−0.23)	−0.2 (−0.25,−0.14)
	R	−0.29 (−0.35,−0.24)	−0.22 (−0.27,−0.17)
Superior temporal	L	−0.36 (−0.41,−0.3)	−0.22 (−0.27,−0.17)
	R	−0.38 (−0.43,−0.32)	−0.23 (−0.29,−0.18)
Supramarginal	L	−0.42 (−0.47,−0.36)	−0.17 (−0.23,−0.12)
	R	−0.39 (−0.44,−0.34)	−0.19 (−0.25,−0.14)
Temporal pole	L	−0.17 (−0.22,−0.12)	−0.09 (−0.14,−0.03)
	R	−0.17 (−0.22,−0.11)	−0.07 (−0.12,−0.01)
Transverse temporal	L	−0.26 (−0.31,−0.2)	−0.15 (−0.21,−0.1)
	R	−0.29 (−0.34,−0.23)	−0.19 (−0.24,−0.14)
Volume			
Accumbens	L	−0.06 (−0.11,−0.01)	
	R	−0.14 (−0.19,−0.09)	
Amygdala	L	−0.25 (−0.3,−0.2)	
	R	−0.24 (−0.3,−0.19)	
Caudate	L	0.03 (−0.03,0.08)	
	R	0.03 (−0.02,0.08)	
Hippocampus	L	−0.43 (−0.48,−0.38)	
	R	−0.42 (−0.48,−0.37)	
Lateral Ventricle	L	0.25 (0.19,0.3)	
	R	0.2 (0.15,0.26)	
Pallidum	L	0.28 (0.23,0.33)	
	R	0.19 (0.14,0.24)	
Putamen	L	0.09 (0.04,0.15)	
	R	0.1 (0.05,0.15)	
Thalamus	L	−0.33 (−0.39,−0.28)	
	R	−0.35 (−0.4,−0.29)	

## CRediT authorship contribution statement

Joaquim Radua: Conceptualization, Data curation, Formal analysis, Funding acquisition, Investigation, Project administration, Supervision, Writing - original draft. Eduard Vieta: Conceptualization, Data curation, Funding acquisition, Investigation, Project administration, Supervision, Writing - review & editing. Russell Shinohara: Data curation, Formal analysis, Writing - review & editing. Peter Kochunov: Conceptualization, Data curation, Formal analysis, Funding acquisition, Project administration, Supervision, Writing - review & editing. Yann Quidé: Data curation, Formal analysis, Investigation, Writing - review & editing. Melissa J. Green: Conceptualization, Data curation, Funding acquisition, Investigation, Project administration, Supervision, Writing - review & editing. Cynthia S. Weickert: Conceptualization, Data curation, Formal analysis, Funding acquisition, Project administration, Supervision, Writing - review & editing. Thomas Weickert: Conceptualization, Data curation, Funding acquisition, Investigation, Project administration, Supervision, Writing - review & editing. Jason Bruggemann: Data curation, Formal analysis, Writing - review & editing. Tilo Kircher: Conceptualization, Funding acquisition, Project administration, Supervision, Writing - review & editing. Igor Nenadić: Conceptualization, Funding acquisition, Project administration, Supervision, Writing - review & editing. Murray J. Cairns: Conceptualization, Data curation, Funding acquisition, Investigation, Project administration, Supervision, Writing - review & editing. Marc Seal: Data curation, Investigation, Writing - review & editing. Ulrich Schall: Data curation, Investigation, Writing - review & editing. Frans Henskens: Data curation, Investigation, Writing - review & editing. Janice M. Fullerton: Data curation, Investigation, Writing - review & editing. Bryan Mowry: Writing - review & editing. Christos Pantelis: Conceptualization, Data curation, Funding acquisition, Investigation, Project administration, Supervision, Writing - review & editing. Rhoshel Lenroot: Data curation, Investigation, Writing - review & editing. Vanessa Cropley: Writing - review & editing. Carmel Loughland: Writing - review & editing. Rodney Scott: Conceptualization, Data curation, Funding acquisition, Investigation, Project administration, Supervision, Writing - review & editing. Daniel Wolf: Data curation, Investigation, Writing - review & editing. Theodore D. Satterthwaite: Conceptualization, Data curation, Formal analysis, Funding acquisition, Investigation, Project administration, Supervision, Writing - review & editing. Yunlong Tan: Data curation, Investigation, Writing - review & editing. Kang Sim: Conceptualization, Data curation, Funding acquisition, Investigation, Project administration, Supervision, Writing - review & editing. Fabrizio Piras: Data curation, Investigation, Writing - review & editing. Gianfranco Spalletta: Conceptualization, Data curation, Funding acquisition, Investigation, Project administration, Supervision, Writing - review & editing. Nerisa Banaj: Data curation, Investigation, Writing - review & editing. Edith Pomarol-Clotet: Conceptualization, Data curation, Funding acquisition, Investigation, Project administration, Supervision, Writing - review & editing. Aleix Solanes: Data curation, Formal analysis, Writing - review & editing. Anton Albajes-Eizagirre: Data curation, Formal analysis, Writing - review & editing. Erick J. Canales-Rodríguez: Data curation, Formal analysis, Investigation, Writing - review & editing. Salvador Sarro: Data curation, Investigation, Writing - review & editing. Annabella Di Giorgio: Conceptualization, Data curation, Formal analysis, Funding acquisition, Project administration, Supervision, Writing - review & editing. Alessandro Bertolino: Data curation, Investigation, Writing - review & editing. Michael Stäblein: Data curation, Investigation, Writing - review & editing. Viola Oertel: Conceptualization, Data curation, Funding acquisition, Investigation, Project administration, Supervision, Writing - review & editing. Christian Knoächel: Data curation, Investigation, Writing - review & editing. Stefan Borgwardt: Conceptualization, Funding acquisition, Project administration, Supervision, Writing - review & editing. Stefan du Plessis: Writing - review & editing. Je-Yeon Yun: Data curation, Formal analysis, Writing - review & editing. Jun Soo Kwon: Conceptualization, Data curation, Funding acquisition, Investigation, Project administration, Supervision, Writing - review & editing. Udo Dannlowski: Conceptualization, Data curation, Funding acquisition, Investigation, Project administration, Supervision, Writing - review & editing. Tim Hahn: Writing - review & editing. Dominik Grotegerd: Data curation, Investigation, Writing - review & editing. Clara Alloza: Data curation, Formal analysis, Writing - review & editing. Celso Arango: Conceptualization, Funding acquisition, Project administration, Supervision, Writing - review & editing. Joost Janssen: Data curation, Formal analysis, Investigation, Writing - review & editing. Covadonga Díaz-Caneja: Data curation, Investigation, Writing - review & editing. Wenhao Jiang: Writing - review & editing. Vince Calhoun: Conceptualization, Data curation, Funding acquisition, Investigation, Project administration, Supervision, Writing - review & editing. Stefan Ehrlich: Conceptualization, Data curation, Formal analysis, Funding acquisition, Project administration, Supervision, Writing - review & editing. Kun Yang: Data curation, Formal analysis, Writing - review & editing. Nicola G. Cascella: Conceptualization, Data curation, Funding acquisition, Investigation, Project administration, Supervision, Writing - review & editing. Yoichiro Takayanagi: Data curation, Investigation, Writing - review & editing. Akira Sawa: Conceptualization, Funding acquisition, Project administration, Supervision, Writing - review & editing. Alexander Tomyshev: Data curation, Formal analysis, Investigation, Writing - review & editing. Irina Lebedeva: Conceptualization, Data curation, Funding acquisition, Investigation, Project administration, Supervision, Writing - review & editing. Vasily Kaleda: Data curation, Formal analysis, Investigation, Writing - review & editing. Matthias Kirschner: Conceptualization, Data curation, Formal analysis, Funding acquisition, Investigation, Project administration, Supervision, Writing - review & editing. Cyril Hoschl: Funding acquisition, Investigation, Writing - review & editing. David Tomecek: Data curation, Formal analysis, Writing - review & editing. Antonin Skoch: Data curation, Formal analysis, Project administration, Supervision, Writing - review & editing. Therese van Amelsvoort: Conceptualization, Data curation, Funding acquisition, Investigation, Project administration, Supervision, Writing - review & editing. Geor Bakker: Data curation, Formal analysis, Investigation, Writing - review & editing. Anthony James: Data curation, Funding acquisition, Investigation, Writing - review & editing. Adrian Preda: Data curation, Investigation, Writing - review & editing. Andrea Weideman: Writing - review & editing. Dan J. Stein: Conceptualization, Funding acquisition, Project administration, Supervision, Writing - review & editing. Fleur Howells: Conceptualization, Funding acquisition, Project administration, Supervision, Writing - review & editing. Anne Uhlmann: Data curation, Formal analysis, Writing - review & editing. Henk Temmingh: Data curation, Investigation, Writing - review & editing. Carlos Loépez-Jaramillo: Conceptualization, Funding acquisition, Project administration, Supervision, Writing - review & editing. Ana Díaz-Zuluaga: Data curation, Investigation, Writing - review & editing. Lydia Fortea: Data curation, Formal analysis, Writing - review & editing. Eloy Martinez-Heras: Writing - review & editing. Elisabeth Solana: Writing - review & editing. Sara Llufriu: Writing - review & editing. Neda Jahanshad: Conceptualization, Funding acquisition, Project administration, Supervision, Writing - review & editing. Paul Thompson: Conceptualization, Funding acquisition, Project administration, Supervision, Writing - review & editing. Jessica Turner: Conceptualization, Funding acquisition, Project administration, Supervision, Writing - review & editing. Theo van Erp: Conceptualization, Data curation, Funding acquisition, Investigation, Project administration, Supervision, Writing - original draft. David Glahn: Conceptualization, Funding acquisition, Project administration, Supervision. Godfrey Pearlson: Conceptualization, Funding acquisition, Project administration, Supervision. Axel Krug: Conceptualization, Funding acquisition, Project administration, Supervision, Writing - review & editing. Vaughan Carr: Conceptualization, Funding acquisition, Project administration, Supervision, Writing - review & editing. Paul Tooney: Conceptualization, Funding acquisition, Project administration, Supervision. Gavin Cooper: Data curation, Investigation, Writing - review & editing. Paul Rasser: Data curation, Investigation, Writing - review & editing. Patricia Michie: Conceptualization, Data curation, Funding acquisition, Investigation, Project administration, Supervision. Fude Yang: Data curation, Investigation. Federica Piras: Data curation, Investigation. Francesca Assogna: Data curation, Investigation. Raymond Salvador: Data curation, Investigation. Peter McKenna: Data curation, Investigation. Aurora Bonvino: Conceptualization, Funding acquisition, Project administration, Supervision. Margaret King: Data curation, Investigation. Stefan Kaiser: Conceptualization, Data curation, Funding acquisition, Investigation, Project administration, Supervision, Writing - review & editing. Dana Nguyen: Data curation, Investigation. Julian Pineda-Zapata: Data curation, Formal analysis.

## ENIGMA Consortium collaborators

David Glahn^ce,cf,cg^

Godfrey Pearlson^cg,ch^

Elliot Hong^j^

Axel Krug^n^

Vaughan Carr^k,ci^

Paul Tooney^o,p^

Gavin Cooper^o^

Paul Rasser^o^

Patricia Michie^o^

Stanley Catts^cj^

Raquel Gur^y^

Ruben Gur^y^

Fude Yang^z^

Fengmei Fan^z^

Jingxu Chen^z^

Hua Guo^ck^

Shuping Tan^z^

Zhiren Wang^z^

Hong Xiang^cl^

Federica Piras^ad^

Francesca Assogna^ad^

Raymond Salvador^b,af^

Peter McKenna^b,af^

Aurora Bonvino^al^

Margaret King^x,cm^

Stefan Kaiser^cn^

Dana Nguyen^co^

Julian Pineda-Zapata^cp^

^a^Imaging of Mood- and Anxiety-Related Disorders (IMARD) Group, Institut d’Investigacions Biomèdiques August Pi i Sunyer (IDIBAPS), Barcelona, Spain

^b^CIBERSAM, Madrid, Spain

^c^Early Psychosis: Interventions and Clinical-detection (EPIC) Lab, Institute of Psychiatry, Psychology and Neuroscience, King’s College London, London, UK

^d^Department of Clinical Neuroscience, Stockholm Health Care Services, Stockholm County Council, Karolinska Institutet, Stockholm, Sweden

^e^Bipolar and depressive disorders, Institut d’Investigacions Biomèdiques August Pi i Sunyer (IDIBAPS), Barcelona, Spain

^f^Barcelona Bipolar Disorders Program, Institute of Neurosciences, Hospital Clinic de Barcelona, Barcelona, Spain

^g^University of Barcelona, Barcelona, Spain

^h^Penn Statistics in Imaging and Visualization Center, Department of Biostatistics, Epidemiology, and Informatics, University of Pennsylvania, Philadelphia, PA, USA

^i^Center for Biomedical Image Computing and Analytics, University of Pennsylvania, Philadelphia, PA, USA

^j^Maryland Psychiatric Research Center, University of Maryland School of Medicine, Baltimore, MD, USA

^k^School of Psychiatry, University of New South Wales, Sydney, NSW, Australia

^l^Neuroscience Research Australia, Sydney, NSW, Australia

^m^Department of Neuroscience & Physiology, Upstate Medical University, Syracuse, Newyork, NY, USA

^n^Department of Psychiatry and Psychotherapy, Philipps-University Marburg, Marburg, Germany

^o^University of Newcastle, Newcastle, NSW, Australia

^p^Hunter Medical Research Institute, Newcastle, NSW, Australia

^q^Murdoch Children’s Research Institute, Melbourne, VIC, Australia

^r^The University of Melbourne, Australia

^s^Health Behaviour Research Group, School of Medicine and Public Health, University of Newcastle, Newcastle, NSW, Australia

^t^Queensland Brain Institute, The University of Queensland, Brisbane, QLD, Australia

^u^Queensland Centre for Mental Health Research, The University of Queensland, Brisbane, QLD, Australia

^v^Melbourne Neuropsychiatry Centre, Dept. of Psychiatry, University of Melbourne, Melbourne, VIC, Australia

^w^North Western Mental Health, Melbourne Health, Melbourne, VIC, Australia

^x^University of New Mexico, Albuquerque, NM, USA

^y^Department of Psychiatry, University of Pennsylvania, Philadelphia, PA, USA

^z^Psychiatry Research Center, Beijing Huilongguan Hospital, Beijing, China

^aa^West Region and Research Division, Institute of Mental Health, Singapore, Singapore

^ab^Yong Loo Lin School of Medicine, National University of Singapore, Singapore, Singapore

^ac^Lee Kong Chian School of Medicine, Nanyang Technological University, Singapore, Singapore

^ad^Laboratory of Neuropsychiatry, Department of Clinical and Behavioral Neurology, IRCCS Santa Lucia Foundation, Rome, Italy

^ae^Division of Neuropsychiatry, Menninger Department of Psychiatry and Behavioral Sciences, Baylor College of Medicine, Houston, TX, USA

^af^FIDMAG Germanes Hospitalàries Research Foundation, Barcelona, Spain

^ag^Department of Psychiatry and Forensic Medicine, School of Medicine, Autonomous University of Barcelona, Barcelona, Spain

^ah^Department of Radiology, Centre Hospitalier Universitaire Vaudois (CHUV), Lausanne, Switzerland

^ai^Signal Processing Lab (LTS5), École Polytechnique Fédérale de Lausanne, Lausanne, Switzerland

^aj^School of Medicine, Universitat Internacional de Catalunya, Barcelona, Spain

^ak^IRCCS Casa Sollievo della Sofferenza, San Giovanni Rotondo, Italy

^al^Department of Basic Medical Science, Neuroscience and Sense Organs, University of Bari ‘Aldo Moro’, Bari, Italy

^am^Dept. of Psychiatry, Psychosomatic Medicine and Psychotherapy, Goethe University Frankfurt, Frankfurt, Germany

^an^Department of Psychiatry, University of Basel, Basel, Switzerland

^ao^University of Stellenbosch, Cape Town, Western Province, South Africa

^ap^Seoul National University Hospital, Seoul, Republic of Korea

^aq^Yeongeon Student Support Center, Seoul National University College of Medicine, Seoul, Republic of Korea

^ar^Department of Psychiatry, Seoul National University College of Medicine, Seoul, Republic of Korea

^as^Department of Brain & Cognitive Sciences, College of Natural Sciences, Seoul National University, Seoul, Republic of Korea

^at^Department of Psychiatry, University of Münster, Münster, Germany

^au^Department of Child and Adolescent Psychiatry, Institute of Psychiatry and Mental Health, Hospital General Universitario Gregorio Marañón, Madrid, Spain

^av^Instituto de Investigación Sanitaria Gregorio Marañón (IiSGM), Madrid, Spain

^aw^School of Medicine, Universidad Complutense, Madrid, Spain

^ax^Georgia State University, Atlanta, GA, USA

^ay^Tri-institutional Center for Translational Research in Neuroimaging and Data Science (TReNDS), Georgia State, Georgia Tech, Emory, Atlanta, GA, USA

^az^Technische Universität Dresden, Faculty of Medicine, Division of Psychological and Social Medicine, Dresden, Germany

^ba^Departments of Psychiatry, Johns Hopkins School of Medicine, Baltimore, MD, USA

^bb^Department of Neuropsychiatry, University of Toyama Graduate School of Medicine and Pharmaceutical Sciences, Toyama, Japan

^bc^Department of Mental Health, Johns Hopkins Bloomberg School of Public Health, Baltimore, MD, USA

^bd^Departments of Psychiatry, Neuroscience, and Biomedical Engineering, Johns Hopkins School of Medicine, Baltimore, MD, USA

^be^School of Medical Sciences, University of New South Wales, Sydney, NSW, Australia

^bf^Mental Health Research Center, Moscow, Russia

^bg^Department of Psychiatry, Psychotherapy and Psychosomatics, Psychiatric Hospital, University of Zurich, Zurich, Switzerland

^bh^Montreal Neurological Institute, McGill University, Montreal, Canada

^bi^National Institute of Mental Health, Klecany, Czech Republic

^bj^Institute of Computer Science, Czech Academy of Sciences, Prague, Czech Republic

^bk^Faculty of Electrical Engineering, Czech Technical University in Prague, Prague, Czech Republic

^bl^MR Unit, Department of Diagnostic and Interventional Radiology, Institute for Clinical and Experimental Medicine, Prague, Czech Republic

^bm^Department of Psychiatry and Neuropsychology, Maastricht University, Maastricht, The Netherlands

^bn^Department of Psychiatry, University of Oxford, Oxford, UK

^bo^Department of Psychiatry and Human Behavior, University of California Irvine, Irvine, CA, USA

^bp^SAMRC Unit on Risk & Resilience in Mental Disorders, Dept of Psychiatry and Neuroscience Institute, University of Cape Town, Cape Town, Western Province, South Africa

^bq^Department of Psychiatry and Mental Health, University of Cape Town, Cape Town, Western Cape, South Africa

^br^Neuroscience Institute, University of Cape Town, Cape Town, Western Cape, South Africa

^bs^Department of Child and Adolescent Psychiatry, Technische Universität Dresden, Dresden, Germany

^bt^Valkenburg Hospital, Observatory, Cape Town, Western Cape, South Africa

^bu^Research Group in Psychiatry GIPSI, Department of Psychiatry, Faculty of Medicine, Universidad de Antioquia, Medellín, Antioquia, Colombia

^bv^Mood Disorders Program, Hospital Universitario San Vicente Fundación, Medellin, Colombia

^bw^Center of Neuroimmunology. Laboratory of Advanced Imaging in Neuroimmunological Diseases. Hospital Clinic de Barcelona, Barcelona, Spain

^bx^Institut d’Investigacions Biomèdiques August Pi i Sunyer (IDIBAPS), Barcelona, Spain

^by^Imaging Genetics Center, Mark & Mary Stevens Neuroimaging & Informatics Institute, Keck School of Medicine, University of Southern California, Los Angeles, CA, USA

^bz^Imaging Genetics Center, Department of Neurology, University of Southern California, Los Angeles, CA, USA

^ca^Clinical Translational Neuroscience Laboratory, Department of Psychiatry and Human Behavior, University of California Irvine, Irvine, CA, USA

^cb^Center for the Neurobiology of Learning and Memory, University of California Irvine, 309 Qureshey Research Lab, Irvine, CA, 92697, USA

^cc^Department of Psychiatry and Psychotherapy, University Lübeck, Germany

^cd^Department of Psychiatry and Clinical Psychology, Third Faculty of Medicine, Charles University, Prague, Czech Republic

^ce^Boston Children's Hospital, Boston, MA, USA

^cf^Harvard Medical School, Boston, MA, USA

^cg^Olin Neuropsychiatry Research Center, Institute of Living, Hartford Hospital, Hartford, CT, USA

^ch^Depts. of Psychiatry and Neuroscience, Yale University, New Haven, CT, USA

^ci^Monash University, Melbourne, VIC, Australia

^cj^University of Queensland, Brisbane, QLD, Australia

^ck^Zhumadian Psychiatry Hospital, Zhumadian, Henan province, China

^cl^Chongqing Three Gorges Central Hospital, Chongqing, China

^cm^Mind Research Network, Albuquerque, NM, USA

^cn^Geneva University Hospitals, Geneva, Switzerland

^co^Department of Pediatrics, University of California Irvine, Irvine, CA, USA

^cp^Research Group, Instituto de Alta Tecnología Médica (IATM), Medellín, Antioquia, Colombia

## References

[R1] Boedhoe PS, Schmaal L, Abe Y, Ameis SH, Arnold PD, Batistuzzo MC, Benedetti F, Beucke JC, Bollettini I, Bose A, Brem S, Calvo A, Cheng Y, Cho KI, Dallaspezia S, Denys D, Fitzgerald KD, Fouche JP, Gimenez M, Gruner P, Hanna GL, Hibar DP, Hoexter MQ, Hu H, Huyser C, Ikari K, Jahanshad N, Kathmann N, Kaufmann C, Koch K, Kwon JS, Lazaro L, Liu Y, Lochner C, Marsh R, Martinez-Zalacain I, Mataix-Cols D, Menchon JM, Minuzzi L, Nakamae T, Nakao T, Narayanaswamy JC, Piras F, Piras F, Pittenger C, Reddy YC, Sato JR, Simpson HB, Soreni N, Soriano-Mas C, Spalletta G, Stevens MC, Szeszko PR, Tolin DF, Venkatasubramanian G, Walitza S, Wang Z, van Wingen GA, Xu J, Xu X, Yun JY, Zhao Q, Group EOW, Thompson PM, Stein DJ, van den Heuvel OA, 2017 Distinct subcortical volume Alterations in pediatric and adult OCD: a worldwide meta- and mega-analysis. Am. J. Psychiatr 174, 60–69. 27609241 10.1176/appi.ajp.2016.16020201 PMC5344782

[R2] Boedhoe PSW, Heymans MW, Schmaal L, Abe Y, Alonso P, Ameis SH, Anticevic A, Arnold PD, Batistuzzo MC, Benedetti F, Beucke JC, Bollettini I, Bose A, Brem S, Calvo A, Calvo R, Cheng Y, Cho KIK, Ciullo V, Dallaspezia S, Denys D, Feusner JD, Fitzgerald KD, Fouche JP, Fridgeirsson EA, Gruner P, Hanna GL, Hibar DP, Hoexter MQ, Hu H, Huyser C, Jahanshad N, James A, Kathmann N, Kaufmann C, Koch K, Kwon JS, Lazaro L, Lochner C, Marsh R, Martinez-Zalacain I, Mataix-Cols D, Menchon JM, Minuzzi L, Morer A, Nakamae T, Nakao T, Narayanaswamy JC, Nishida S, Nurmi EL, O’Neill J, Piacentini J, Piras F, Piras F, Reddy YCJ, Reess TJ, Sakai Y, Sato JR, Simpson HB, Soreni N, Soriano-Mas C, Spalletta G, Stevens MC, Szeszko PR, Tolin DF, van Wingen GA, Venkatasubramanian G, Walitza S, Wang Z, Yun JY, Working- Group, E.-O., Thompson PM, Stein DJ, van den Heuvel OA, Twisk JWR, 2018 An empirical comparison of meta- and mega-analysis with data from the ENIGMA obsessive-compulsive disorder working group. Front. Neuroinf 12, 102. 10.3389/fninf.2018.00102 PMC6331928 30670959

[R3] Favre P, Pauling M, Stout J, Hozer F, Sarrazin S, Abe C, Alda M, Alloza C, Alonso-Lana S, Andreassen OA, Baune BT, Benedetti F, Busatto GF, Canales-Rodriguez EJ, Caseras X, Chaim-Avancini TM, Ching CRK, Dannlowski U, Deppe M, Eyler LT, Fatjo-Vilas M, Foley SF, Grotegerd D, Hajek T, Haukvik UK, Howells FM, Jahanshad N, Kugel H, Lagerberg TV, Lawrie SM, Linke JO, McIntosh A, Melloni EMT, Mitchell PB, Polosan M, Pomarol-Clotet E, Repple J, Roberts G, Roos A, Rosa PGP, Salvador R, Sarro S, Schofield PR, Serpa MH, Sim K, Stein DJ, Sussmann JE, Temmingh HS, Thompson PM, Verdolini N, Vieta E, Wessa M, Whalley HC, Zanetti MV, Leboyer M, Mangin JF, Henry C, Duchesnay E, Houenou J, Group EBDW, 2019 Widespread white matter microstructural abnormalities in bipolar disorder: evidence from mega- and meta-analyses across 3033 individuals. Neuropsychopharmacology 44, 2285–2293. 31434102 10.1038/s41386-019-0485-6 PMC6898371

[R4] Thompson PM, Stein JL, Medland SE, Hibar DP, Vasquez AA, Renteria ME, Toro R, Jahanshad N, Schumann G, Franke B, Wright MJ, Martin NG, Agartz I, Alda M, Alhusaini S, Almasy L, Almeida J, Alpert K, Andreasen NC, Andreassen OA, Apostolova LG, Appel K, Armstrong NJ, Aribisala B, Bastin ME, Bauer M, Bearden CE, Bergmann O, Binder EB, Blangero J, Bockholt HJ, Boen E, Bois C, Boomsma DI, Booth T, Bowman IJ, Bralten J, Brouwer RM, Brunner HG, Brohawn DG, Buckner RL, Buitelaar J, Bulayeva K, Bustillo JR, Calhoun VD, Cannon DM, Cantor RM, Carless MA, Caseras X, Cavalleri GL, Chakravarty MM, Chang KD, Ching CR, Christoforou A, Cichon S, Clark VP, Conrod P, Coppola G, Crespo-Facorro B, Curran JE, Czisch M, Deary IJ, de Geus EJ, den Braber A, Delvecchio G, Depondt C, de Haan L, de Zubicaray GI, Dima D, Dimitrova R, Djurovic S, Dong H, Donohoe G, Duggirala R, Dyer TD, Ehrlich S, Ekman CJ, Elvsashagen T, Emsell L, Erk S, Espeseth T, Fagerness J, Fears S, Fedko I, Fernandez G, Fisher SE, Foroud T, Fox PT, Francks C, Frangou S, Frey EM, Frodl T, Frouin V, Garavan H, Giddaluru S, Glahn DC, Godlewska B, Goldstein RZ, Gollub RL, Grabe HJ, Grimm O, Gruber O, Guadalupe T, Gur RE, Gur RC, Goring HH, Hagenaars S, Hajek T, Hall GB, Hall J, Hardy J, Hartman CA, Hass J, Hatton SN, Haukvik UK, Hegenscheid K, Heinz A, Hickie IB, Ho BC, Hoehn D, Hoekstra PJ, Hollinshead M, Holmes AJ, Homuth G, Hoogman M, Hong LE, Hosten N, Hottenga JJ, Hulshoff Pol HE, Hwang KS, Jack CR Jr., Jenkinson M, Johnston C, Jonsson EG, Kahn RS, Kasperaviciute D, Kelly S, Kim S, Kochunov P, Koenders L, Kramer B, Kwok JB, Lagopoulos J, Laje G, Landen M, Landman BA, Lauriello J, Lawrie SM, Lee PH, Le Hellard S, Lemaitre H, Leonardo CD, Li CS, Liberg B, Liewald DC, Liu X, Lopez LM, Loth E, Lourdusamy A, Luciano M, Macciardi F, Machielsen MW, Macqueen GM, Malt UF, Mandl R, Manoach DS, Martinot JL, Matarin M, Mather KA, Mattheisen M, Mattingsdal M, Meyer-Lindenberg A, McDonald C, McIntosh AM, McMahon FJ, McMahon KL, Meisenzahl E, Melle I, Milaneschi Y, Mohnke S, Montgomery GW, Morris DW, Moses EK, Mueller BA, Munoz Maniega S, Muhleisen TW, Muller-Myhsok B, Mwangi B, Nauck M, Nho K, Nichols TE, Nilsson LG, Nugent AC, Nyberg L, Olvera RL, Oosterlaan J, Ophoff RA, Pandolfo M, Papalampropoulou-Tsiridou M, Papmeyer M, Paus T, Pausova Z, Pearlson GD, Penninx BW, Peterson CP, Pfennig A, Phillips M, Pike GB, Poline JB, Potkin SG, Putz B, Ramasamy A, Rasmussen J, Rietschel M, Rijpkema M, Risacher SL, Roffman JL, Roiz-Santianez R, Romanczuk-Seiferth N, Rose EJ, Royle NA, Rujescu D, Ryten M, Sachdev PS, Salami A, Satterthwaite TD, Savitz J, Saykin AJ, Scanlon C, Schmaal L, Schnack HG, Schork AJ, Schulz SC, Schur R, Seidman L, Shen L, Shoemaker JM, Simmons A, Sisodiya SM, Smith C, Smoller JW, Soares JC, Sponheim SR, Sprooten E, Starr JM, Steen VM, Strakowski S, Strike L, Sussmann J, Samann PG, Teumer A, Toga AW, Tordesillas-Gutierrez D, Trabzuni D, Trost S, Turner J, Van den Heuvel M, van der Wee NJ, van Eijk K, van Erp TG, van Haren NE, van ‘t Ent D, van Tol MJ, Valdes Hernandez MC, Veltman DJ, Versace A, Volzke H, Walker R, Walter H, Wang L, Wardlaw JM, Weale ME, Weiner MW, Wen W, Westlye LT, Whalley HC, Whelan CD, White T, Winkler AM, Wittfeld K, Woldehawariat G, Wolf C, Zilles D, Zwiers MP, Thalamuthu A, Schofield PR, Freimer NB, Lawrence NS, Drevets W, Alzheimer’s Disease Neuroimaging Initiative, E.C.I.C.S.Y.S.G, 2014 The ENIGMA Consortium: large-scale collaborative analyses of neuroimaging and genetic data. Brain Imaging Behav 8, 153–182. 24399358 10.1007/s11682-013-9269-5 PMC4008818

[R5] van Erp TG, Hibar DP, Rasmussen JM, Glahn DC, Pearlson GD, Andreassen OA, Agartz I, Westlye LT, Haukvik UK, Dale AM, Melle I, Hartberg CB, Gruber O, Kraemer B, Zilles D, Donohoe G, Kelly S, McDonald C, Morris DW, Cannon DM, Corvin A, Machielsen MW, Koenders L, de Haan L, Veltman DJ, Satterthwaite TD, Wolf DH, Gur RC, Gur RE, Potkin SG, Mathalon DH, Mueller BA, Preda A, Macciardi F, Ehrlich S, Walton E, Hass J, Calhoun VD, Bockholt HJ, Sponheim SR, Shoemaker JM, van Haren NE, Hulshoff Pol HE, Ophoff RA, Kahn RS, Roiz-Santianez R, Crespo-Facorro B, Wang L, Alpert KI, Jonsson EG, Dimitrova R, Bois C, Whalley HC, McIntosh AM, Lawrie SM, Hashimoto R, Thompson PM, Turner JA, 2016 Subcortical brain volume abnormalities in 2028 individuals with schizophrenia and 2540 healthy controls via the ENIGMA consortium. Mol. Psychiatr 21, 547–553. 10.1038/mp.2015.63 PMC4668237 26033243

[R6] van Erp TGM, Walton E, Hibar DP, Schmaal L, Jiang W, Glahn DC, Pearlson GD, Yao N, Fukunaga M, Hashimoto R, Okada N, Yamamori H, Bustillo JR, Clark VP, Agartz I, Mueller BA, Cahn W, de Zwarte SMC, Hulshoff Pol HE, Kahn RS, Ophoff RA, van Haren NEM, Andreassen OA, Dale AM, Doan NT, Gurholt TP, Hartberg CB, Haukvik UK, Jorgensen KN, Lagerberg TV, Melle I, Westlye LT, Gruber O, Kraemer B, Richter A, Zilles D, Calhoun VD, Crespo-Facorro B, Roiz-Santianez R, Tordesillas-Gutierrez D, Loughland C, Carr VJ, Catts S, Cropley VL, Fullerton JM, Green MJ, Henskens FA, Jablensky A, Lenroot RK, Mowry BJ, Michie PT, Pantelis C, Quide Y, Schall U, Scott RJ, Cairns MJ, Seal M, Tooney PA, Rasser PE, Cooper G, Shannon Weickert C, Weickert TW, Morris DW, Hong E, Kochunov P, Beard LM, Gur RE, Gur RC, Satterthwaite TD, Wolf DH, Belger A, Brown GG, Ford JM, Macciardi F, Mathalon DH, O’Leary DS, Potkin SG, Preda A, Voyvodic J, Lim KO, McEwen S, Yang F, Tan Y, Tan S, Wang Z, Fan F, Chen J, Xiang H, Tang S, Guo H, Wan P, Wei D, Bockholt HJ, Ehrlich S, Wolthusen RPF, King MD, Shoemaker JM, Sponheim SR, De Haan L, Koenders L, Machielsen MW, van Amelsvoort T, Veltman DJ, Assogna F, Banaj N, de Rossi P, Iorio M, Piras F, Spalletta G, McKenna PJ, Pomarol-Clotet E, Salvador R, Corvin A, Donohoe G, Kelly S, Whelan CD, Dickie EW, Rotenberg D, Voineskos AN, Ciufolini S, Radua J, Dazzan P, Murray R, Reis Marques T, Simmons A, Borgwardt S, Egloff L, Harrisberger F, Riecher-Rossler A, Smieskova R, Alpert KI, Wang L, Jonsson EG, Koops S, Sommer IEC, Bertolino A, Bonvino A, Di Giorgio A, Neilson E, Mayer AR, Stephen JM, Kwon JS, Yun JY, Cannon DM, McDonald C, Lebedeva I, Tomyshev AS, Akhadov T, Kaleda V, Fatouros-Bergman H, Flyckt L, Karolinska Schizophrenia P, Busatto GF, Rosa PGP, Serpa MH, Zanetti MV, Hoschl C, Skoch A, Spaniel F, Tomecek D, Hagenaars SP, McIntosh AM, Whalley HC, Lawrie SM, Knochel C, Oertel-Knochel V, Stablein M, Howells FM, Stein DJ, Temmingh HS, Uhlmann A, Lopez-Jaramillo C, Dima D, McMahon A, Faskowitz JI, Gutman BA, Jahanshad N, Thompson PM, Turner JA, 2018 Cortical brain abnormalities in 4474 individuals with schizophrenia and 5098 control subjects via the enhancing Neuro imaging Genetics through meta analysis (ENIGMA) consortium. Biol. Psychiatr 84, 644–654. 10.1016/j.biopsych.2018.04.023 PMC6177304 29960671

[R7] van Rooij D, Anagnostou E, Arango C, Auzias G, Behrmann M, Busatto GF, Calderoni S, Daly E, Deruelle C, Di Martino A, Dinstein I, Duran FLS, Durston S, Ecker C, Fair D, Fedor J, Fitzgerald J, Freitag CM, Gallagher L, Gori I, Haar S, Hoekstra L, Jahanshad N, Jalbrzikowski M, Janssen J, Lerch J, Luna B, Martinho MM, McGrath J, Muratori F, Murphy CM, Murphy DGM, O’Hearn K, Oranje B, Parellada M, Retico A, Rosa P, Rubia K, Shook D, Taylor M, Thompson PM, Tosetti M, Wallace GL, Zhou F, Buitelaar JK, 2018 Cortical and subcortical brain morphometry differences between patients with autism spectrum disorder and healthy individuals across the lifespan: results from the ENIGMA ASD working group. Am. J. Psychiatr 175, 359–369. 29145754 10.1176/appi.ajp.2017.17010100 PMC6546164

[R8] Wong TY, Radua J, Pomarol-Clotet E, Salvador R, Albajes-Eizagirre A, Solanes A, Canales-Rodriguez EJ, Guerrero-Pedraza A, Sarro S, Kircher T, Nenadic I, Krug A, Grotegerd D, Dannlowski U, Borgwardt S, Riecher-Rossler A, Schmidt A, Andreou C, Huber CG, Turner J, Calhoun V, Jiang W, Clark S, Walton E, Spalletta G, Banaj N, Piras F, Ciullo V, Vecchio D, Lebedeva I, Tomyshev AS, Kaleda V, Klushnik T, Filho GB, Zanetti MV, Serpa MH, Penteado Rosa PG, Hashimoto R, Fukunaga M, Richter A, Kramer B, Gruber O, Voineskos AN, Dickie EW, Tomecek D, Skoch A, Spaniel F, Hoschl C, Bertolino A, Bonvino A, Di Giorgio A, Holleran L, Ciufolini S, Marques TR, Dazzan P, Murray R, Lamsma J, Cahn W, van Haren N, Diaz-Zuluaga AM, Pineda-Zapata JA, Vargas C, Lopez-Jaramillo C, van Erp TGM, Gur RC, Nickl-Jockschat T, 2019 An overlapping pattern of cerebral cortical thinning is associated with both positive symptoms and aggression in schizophrenia via the ENIGMA consortium. Psychol. Med 1–12. 10.1017/S0033291719002149 PMC13196894 31615588

[R9] Albajes-Eizagirre A, Radua J, 2018 What do results from coordinate-based meta-analyses tell us? Neuroimage 176, 550–553. 29729389 10.1016/j.neuroimage.2018.04.065

[R10] Albajes-Eizagirre A, Solanes A, Vieta E, Radua J, 2019 Voxel-based meta-analysis via permutation of subject images (PSI): theory and implementation for SDM. Neuroimage 186, 174–184. 30389629 10.1016/j.neuroimage.2018.10.077

[R11] Bates D, Maechler M, Bolker B, Walker S, 2015 Fitting linear mixed-effects models using lme4. J. Stat. Software 67, 1–48.

[R12] Blakesley RE, Mazumdar S, Dew MA, Houck PR, Tang G, Reynolds CF 3rd, Butters MA, 2009 Comparisons of methods for multiple hypothesis testing in neuropsychological research. Neuropsychology 23, 255–264. 19254098 10.1037/a0012850 PMC3045855

[R13] Chen B, Benedetti A, 2017 Quantifying heterogeneity in individual participant data meta-analysis with binary outcomes. Syst. Rev 6, 243. 29208048 10.1186/s13643-017-0630-4 PMC5718085

[R14] Chepkoech JL, Walhovd KB, Grydeland H, Fjell AM, Alzheimer’s Disease Neuroimaging I, 2016 Effects of change in FreeSurfer version on classification accuracy of patients with Alzheimer’s disease and mild cognitive impairment. Hum. Brain Mapp 37, 1831–1841. 27018380 10.1002/hbm.23139 PMC6867543

[R15] Dale AM, Fischl B, Sereno MI, 1999 Cortical surface-based analysis. I. Segmentation and surface reconstruction. Neuroimage 9, 179–194. 9931268 10.1006/nimg.1998.0395

[R16] Desikan RS, Segonne F, Fischl B, Quinn BT, Dickerson BC, Blacker D, Buckner RL, Dale AM, Maguire RP, Hyman BT, Albert MS, Killiany RJ, 2006 An automated labeling system for subdividing the human cerebral cortex on MRI scans into gyral based regions of interest. Neuroimage 31, 968–980. 16530430 10.1016/j.neuroimage.2006.01.021

[R17] Eickhoff SB, Laird AR, Grefkes C, Wang LE, Zilles K, Fox PT, 2009 Coordinate-based activation likelihood estimation meta-analysis of neuroimaging data: a random-effects approach based on empirical estimates of spatial uncertainty. Hum. Brain Mapp 30, 2907–2926. 19172646 10.1002/hbm.20718 PMC2872071

[R18] Eickhoff SB, Bzdok D, Laird AR, Kurth F, Fox PT, 2012 Activation likelihood estimation meta-analysis revisited. Neuroimage 59, 2349–2361. 21963913 10.1016/j.neuroimage.2011.09.017 PMC3254820

[R19] Fischl B, 2012 FreeSurfer. Neuroimage 62, 774–781. 22248573 10.1016/j.neuroimage.2012.01.021 PMC3685476

[R20] Fischl B, Sereno MI, Dale AM, 1999 Cortical surface-based analysis. II: inflation, flattening, and a surface-based coordinate system. Neuroimage 9, 195–207. 9931269 10.1006/nimg.1998.0396

[R21] Fortin JP, Parker D, Tunc B, Watanabe T, Elliott MA, Ruparel K, Roalf DR, Satterthwaite TD, Gur RC, Gur RE, Schultz RT, Verma R, Shinohara RT, 2017 Harmonization of multi-site diffusion tensor imaging data. Neuroimage 161, 149–170. 28826946 10.1016/j.neuroimage.2017.08.047 PMC5736019

[R22] Fortin JP, Cullen N, Sheline YI, Taylor WD, Aselcioglu I, Cook PA, Adams P, Cooper C, Fava M, McGrath PJ, McInnis M, Phillips ML, Trivedi MH, Weissman MM, Shinohara RT, 2018 Harmonization of cortical thickness measurements across scanners and sites. Neuroimage 167, 104–120. 29155184 10.1016/j.neuroimage.2017.11.024 PMC5845848

[R23] Gronenschild EH, Habets P, Jacobs HI, Mengelers R, Rozendaal N, van Os J, Marcelis M, 2012 The effects of FreeSurfer version, workstation type, and Macintosh operating system version on anatomical volume and cortical thickness measurements. PLoS One 7, e38234. 22675527 10.1371/journal.pone.0038234 PMC3365894

[R24] Holm S, 1979 A simple sequentially rejective multiple test procedure. Scand. J. Stat 6, 65–70.

[R25] Huber G, 1957 Pneumencephalographische und psychopathologische Bilder bei endogenen Psychosen. Springer, Berlin, Heidelberg.

[R26] Johnson WE, Li C, Rabinovic A, 2007 Adjusting batch effects in microarray expression data using empirical Bayes methods. Biostatistics 8, 118–127. 16632515 10.1093/biostatistics/kxj037

[R27] Kay SR, Fiszbein A, Opler LA, 1987 The positive and negative syndrome scale (PANSS) for schizophrenia. Schizophr. Bull 13, 261–276. 3616518 10.1093/schbul/13.2.261

[R28] Kuznetsova A, Brockhoff PB, Christensen RHB, 2017 lmerTest package: tests in linear mixed effects models. J. Stat. Software 82, 1–26.

[R29] Leek JT, Johnson WE, Parker HS, Fertig EJ, Jaffe AE, Storey JD, Zhang Y, Torres LC, 2019 Sva: Surrogate Variable Analysis. R package.

[R30] Radua J, Mataix-Cols D, 2012 Meta-analytic methods for neuroimaging data explained. Biol. Mood Anxiety Disord 2, 6. 22737993 10.1186/2045-5380-2-6 PMC3384225

[R31] Radua J, Mataix-Cols D, Phillips ML, El-Hage W, Kronhaus DM, Cardoner N, Surguladze S, 2012 A new meta-analytic method for neuroimaging studies that combines reported peak coordinates and statistical parametric maps. Eur. Psychiatr 27, 605–611. 10.1016/j.eurpsy.2011.04.001 21658917

[R32] Tustison NJ, Cook PA, Klein A, Song G, Das SR, Duda JT, Kandel BM, van Strien N, Stone JR, Gee JC, Avants BB, 2014 Large-scale evaluation of ANTs and FreeSurfer cortical thickness measurements. Neuroimage 99, 166–179. 24879923 10.1016/j.neuroimage.2014.05.044

[R33] Viechtbauer W, 2010 Conducting meta-analyses in R with the metafor package. J. Stat. Software 36, 1–48.

[R34] Winkler AM, Ridgway GR, Webster MA, Smith SM, Nichols TE, 2014 Permutation inference for the general linear model. Neuroimage 92, 381–397. 24530839 10.1016/j.neuroimage.2014.01.060 PMC4010955

[R35] Yu M, Linn KA, Cook PA, Phillips ML, McInnis M, Fava M, Trivedi MH, Weissman MM, Shinohara RT, Sheline YI, 2018 Statistical harmonization corrects site effects in functional connectivity measurements from multi-site fMRI data. Hum. Brain Mapp 39, 4213–4227. 29962049 10.1002/hbm.24241 PMC6179920

