# Supplementary material for: Increased power by harmonizing structural MRI site differences with the ComBat batch adjustment method in ENIGMA
Source: Neuroimage. Author manuscript; Available in PMC 2020 Sep 29. (PMC7524039; doi:10.1016/j.neuroimage.2020.116956)
Supplement: 1 [file NIHMS1619222-supplement-1.docx]

Increased power by harmonizing structural MRI site differences

with the ComBat batch adjustment method in ENIGMA

**SUPPLEMENT**

**Supplementary Table S1**. Description of the sample of each site.

| Site | Sample | Size | Age  (SD) | Females | Age of onset  (SD) | Duration of illness  (SD) | PANSS | | | SAPS  (SD) | SANS  (SD) | CDE  (SD) |
| --- | --- | --- | --- | --- | --- | --- | --- | --- | --- | --- | --- | --- |
|  |  |  |  |  |  |  | Total  (SD) | Positive  (SD) | Negative  (SD) |  |  |  |
| ASRB1 | Patients with schizophrenia | 109 | 38.6 | 27.5% | 24.1 | 14.6 |  |  |  |  | 15.8 |  |
|  |  |  | 11.3 |  | 7.3 | 11.1 |  |  |  |  | 14.3 |  |
|  | Healthy controls | 34 | 40.5 | 55.9% |  |  |  |  |  |  |  |  |
|  |  |  | 14.1 |  |  |  |  |  |  |  |  |  |
| ASRB2 | Patients with schizophrenia | 56 | 37.5 | 35.7% | 23.5 | 13.9 |  |  |  |  | 21.3 |  |
|  |  |  | 9.6 |  | 6.4 | 8.4 |  |  |  |  | 12.9 |  |
|  | Healthy controls | 62 | 38.2 | 50.0% |  |  |  |  |  |  |  |  |
|  |  |  | 13.1 |  |  |  |  |  |  |  |  |  |
| ASRB3 | Patients with schizophrenia | 20 | 38.4 | 40.0% | 23.4 | 15.1 |  |  |  |  | 6 |  |
|  |  |  | 9.1 |  | 6.3 | 6.9 |  |  |  |  | 7 |  |
|  | Healthy controls | 13 | 43.1 | 46.2% |  |  |  |  |  |  |  |  |
|  |  |  | 13 |  |  |  |  |  |  |  |  |  |
| ASRB4 | Patients with schizophrenia | 24 | 37.4 | 41.7% | 22.7 | 14.7 |  |  |  |  | 14.5 |  |
|  |  |  | 10.6 |  | 5 | 9.2 |  |  |  |  | 11.3 |  |
|  | Healthy controls | 25 | 37.2 | 52.0% |  |  |  |  |  |  |  |  |
|  |  |  | 14.4 |  |  |  |  |  |  |  |  |  |
| ASRB5 | Patients with schizophrenia | 54 | 40.3 | 33.3% | 23.3 | 16.9 |  |  |  |  | 27.4 |  |
|  |  |  | 9.3 |  | 6 | 8.5 |  |  |  |  | 15.2 |  |
|  | Healthy controls | 32 | 40.1 | 56.2% |  |  |  |  |  |  |  |  |
|  |  |  | 14 |  |  |  |  |  |  |  |  |  |
| CASSI | Patients with schizophrenia | 58 | 35 | 32.8% | 23 | 12 | 34.6 | 8.6 | 8.9 |  |  | 572.5 |
|  |  |  | 8.8 |  | 5.7 | 7.3 | 17.2 | 5 | 6.3 |  |  | 488.8 |
|  | Healthy controls | 65 | 30.3 | 46.2% |  |  |  |  |  |  |  |  |
|  |  |  | 7.1 |  |  |  |  |  |  |  |  |  |
| CIAM | Patients with schizophrenia | 21 | 31 | 38.1% | 22.8 | 8.3 | 55.5 | 13.6 | 15.2 |  |  |  |
|  |  |  | 6.4 |  | 5.6 | 6.8 | 19.1 | 6.9 | 6.6 |  |  |  |
|  | Healthy controls | 30 | 26.6 | 46.7% |  |  |  |  |  |  |  |  |
|  |  |  | 5 |  |  |  |  |  |  |  |  |  |
| COBRE | Patients with schizophrenia | 73 | 37.4 | 17.8% | 21.4 | 15.8 | 60 | 15.2 | 14.8 |  |  | 547.2 |
|  |  |  | 13.4 |  | 8.2 | 12.6 | 14.9 | 5.1 | 4.7 |  |  | 1079.6 |
|  | Healthy controls | 70 | 35.7 | 28.6% |  |  |  |  |  |  |  |  |
|  |  |  | 11.8 |  |  |  |  |  |  |  |  |  |
| EONKCS | Patients with schizophrenia | 108 | 34.2 | 31.5% | 20.9 | 13.2 |  |  |  | 23.2 | 33 |  |
|  |  |  | 12.9 |  | 7.8 | 12.6 |  |  |  | 17.3 | 18.4 |  |
|  | Healthy controls | 92 | 31.9 | 44.6% |  |  |  |  |  |  |  |  |
|  |  |  | 14.4 |  |  |  |  |  |  |  |  |  |
| ESO | Patients with schizophrenia | 40 | 29.4 | 50.0% | 28.8 | 0.6 | 63.8 | 14.2 | 16.1 |  |  |  |
|  |  |  | 7 |  | 6.9 | 0.8 | 17.4 | 5.6 | 5.1 |  |  |  |
|  | Healthy controls | 40 | 29.1 | 50.0% |  |  |  |  |  |  |  |  |
|  |  |  | 6.5 |  |  |  |  |  |  |  |  |  |
| FIDMAG | Patients with schizophrenia | 160 | 39.6 | 22.5% | 23.3 | 15.4 | 76.5 | 16.7 | 22.9 |  |  | 634.6 |
|  |  |  | 11.8 |  | 7.4 | 11.4 | 17.6 | 5.5 | 6.7 |  |  | 790.5 |
|  | Healthy controls | 123 | 37.5 | 56.1% |  |  |  |  |  |  |  |  |
|  |  |  | 10.1 |  |  |  |  |  |  |  |  |  |
| FOR2107  Marburg | Patients with schizophrenia | 37 | 37.2 | 37.8% | 21.2 | 15.9 |  |  |  | 13.2 | 18.8 | 403.7 |
|  |  |  | 11.7 |  | 6.4 | 10.8 |  |  |  | 13.4 | 13.5 | 409.2 |
|  | Healthy controls | 366 | 34 | 60.9% |  |  |  |  |  |  |  |  |
|  |  |  | 12.7 |  |  |  |  |  |  |  |  |  |
| FOR2107  Muenster | Patients with schizophrenia | 8 | 33.4 | 50.0% | 22.2 | 11.1 |  |  |  | 6.4 | 8.1 | 306.7 |
|  |  |  | 8.7 |  | 4.9 | 9.8 |  |  |  | 9.8 | 10.4 | 195.5 |
|  | Healthy controls | 155 | 27 | 61.3% |  |  |  |  |  |  |  |  |
|  |  |  | 9.5 |  |  |  |  |  |  |  |  |  |
| Frankfurt | Patients with schizophrenia | 124 | 27.4 | 29.8% |  |  | 62.3 | 15.7 | 16.4 |  |  | 198.8 |
|  |  |  | 7.2 |  |  |  | 14.4 | 6.5 | 6 |  |  | 101.6 |
|  | Healthy controls | 88 | 25.9 | 62.5% |  |  |  |  |  |  |  |  |
|  |  |  | 6.4 |  |  |  |  |  |  |  |  |  |
| GIPSI | Patients with schizophrenia | 43 | 33.5 | 18.6% | 19.1 | 14.1 |  |  |  | 9.3 | 32.2 | 423 |
|  |  |  | 11.5 |  | 5.6 | 9.5 |  |  |  | 11.8 | 18.5 | 328.2 |
|  | Healthy controls | 0 |  |  |  |  |  |  |  |  |  |  |
|  |  |  |  |  |  |  |  |  |  |  |  |  |
| Huilong1 | Patients with schizophrenia | 155 | 26.2 | 45.8% |  |  |  |  |  |  |  |  |
|  |  |  | 5.8 |  |  |  |  |  |  |  |  |  |
|  | Healthy controls | 38 | 31.6 | 44.7% |  |  |  |  |  |  |  |  |
|  |  |  | 6.5 |  |  |  |  |  |  |  |  |  |
| Huilong3 | Patients with schizophrenia | 90 | 24.3 | 45.6% |  |  |  |  |  |  |  |  |
|  |  |  | 6.1 |  |  |  |  |  |  |  |  |  |
|  | Healthy controls | 50 | 24.8 | 44.0% |  |  |  |  |  |  |  |  |
|  |  |  | 5.4 |  |  |  |  |  |  |  |  |  |
| IGP | Patients with schizophrenia | 68 | 41.7 | 41.2% | 22.9 | 18.8 | 55.5 | 13.8 | 14.5 | 17.2 | 29.1 | 655.2 |
|  |  |  | 11.1 |  | 7 | 9.5 | 18.4 | 5.7 | 6.1 | 14.8 | 17.9 | 1119.7 |
|  | Healthy controls | 71 | 36 | 45.1% |  |  |  |  |  |  |  |  |
|  |  |  | 11 |  |  |  |  |  |  |  |  |  |
| JHU | Patients with schizophrenia | 49 | 41.2 | 26.5% | 23.8 | 17 |  |  |  | 1.3 | 2.1 |  |
|  |  |  | 11.5 |  | 7.9 | 11.2 |  |  |  | 1 | 1.3 |  |
|  | Healthy controls | 88 | 44.2 | 52.3% |  |  |  |  |  |  |  |  |
|  |  |  | 12.5 |  |  |  |  |  |  |  |  |  |
| Madrid | Patients with schizophrenia | 36 | 16.3 | 18.2% | 15.9 | 28.6 | 22.8 | 24.5 | 24.5 |  |  |  |
|  |  |  | 1.8 |  | 1.7 | 43 | 9.5 | 8.1 | 8.1 |  |  |  |
|  | Healthy controls | 112 | 12.5 | 29.8% |  |  |  |  |  |  |  |  |
|  |  |  | 3.2 |  |  |  |  |  |  |  |  |  |
| MPRC | Patients with schizophrenia | 230 | 36.4 | 38.7% |  |  |  |  |  |  |  |  |
|  |  |  | 13.2 |  |  |  |  |  |  |  |  |  |
|  | Healthy controls | 270 | 37.1 | 56.3% |  |  |  |  |  |  |  |  |
|  |  |  | 15 |  |  |  |  |  |  |  |  |  |
| OLIN | Patients with schizophrenia | 312 | 37.7 | 44.2% |  |  |  |  |  |  |  |  |
|  |  |  | 13.6 |  |  |  |  |  |  |  |  |  |
|  | Healthy controls | 557 | 37.6 | 44.2% |  |  |  |  |  |  |  |  |
|  |  |  | 12.7 |  |  |  |  |  |  |  |  |  |
| Oxford | Patients with schizophrenia | 41 | 16.2 | 41.5% | 14.5 | 1.8 |  | 22.2 | 16.2 |  |  | 353.8 |
|  |  |  | 1.2 |  | 1.5 | 1.3 |  | 2.9 | 3.1 |  |  | 222.5 |
|  | Healthy controls | 33 | 16.1 | 54.5% |  |  |  |  |  |  |  |  |
|  |  |  | 1.4 |  |  |  |  |  |  |  |  |  |
| RomeSL | Patients with schizophrenia | 172 | 39.2 | 32.0% | 24.3 | 14.9 | 87.6 | 21.1 | 21 | 32.3 | 31.5 | 385.1 |
|  |  |  | 11.4 |  | 8.4 | 10.7 | 20.3 | 6.3 | 7.5 | 19.7 | 16.8 | 442.4 |
|  | Healthy controls | 116 | 37.5 | 37.1% |  |  |  |  |  |  |  |  |
|  |  |  | 11.5 |  |  |  |  |  |  |  |  |  |
| RSCZ | Patients with schizophrenia | 45 | 22.1 | 0% | 21 | 1.1 | 60.3 | 11.3 | 18.7 |  |  |  |
|  |  |  | 3.3 |  | 3.1 | 1.3 | 10.7 | 2.9 | 4.6 |  |  |  |
|  | Healthy controls | 49 | 22.2 | 0% |  |  |  |  |  |  |  |  |
|  |  |  | 2.8 |  |  |  |  |  |  |  |  |  |
| SCORE | Patients with schizophrenia | 161 | 25.5 | 27.3% | 24.5 | 1.1 |  |  |  |  | 15.8 | 203.2 |
|  |  |  | 6.1 |  | 5.9 | 2.3 |  |  |  |  | 12.8 | 250.8 |
|  | Healthy controls | 44 | 25.5 | 61.4% |  |  |  |  |  |  |  |  |
|  |  |  | 4.3 |  |  |  |  |  |  |  |  |  |
| Singapore | Patients with schizophrenia | 178 | 33.7 | 32.6% | 25.6 | 7.5 | 39.4 | 10.3 | 9 |  |  | 204.7 |
|  |  |  | 9.4 |  | 7.5 | 8 | 8 | 3.7 | 3 |  |  | 179.9 |
|  | Healthy controls | 111 | 33.3 | 40.5% |  |  |  |  |  |  |  |  |
|  |  |  | 10 |  |  |  |  |  |  |  |  |  |
| SNUH | Patients with schizophrenia | 40 | 22.9 | 55.0% | 22.3 | 6 | 68.6 | 16.6 | 17.2 |  |  | 188.1 |
|  |  |  | 5.6 |  | 5.6 | 4 | 14.8 | 5.2 | 5.7 |  |  | 194.7 |
|  | Healthy controls | 40 | 22.6 | 50.0% |  |  |  |  |  |  |  |  |
|  |  |  | 3.9 |  |  |  |  |  |  |  |  |  |
| UCISZ | Patients with schizophrenia | 26 | 43.4 | 19.2% | 25.2 | 17.8 | 60 | 15.5 | 16.2 | 13.5 | 23.3 |  |
|  |  |  | 10.5 |  | 7.9 | 10.1 | 12.2 | 4.2 | 6 | 10.5 | 14.8 |  |
|  | Healthy controls | 30 | 41.4 | 23.3% |  |  |  |  |  |  |  |  |
|  |  |  | 12.3 |  |  |  |  |  |  |  |  |  |
| UNIBA | Patients with schizophrenia | 88 | 33.6 | 26.1% | 20.6 | 12.1 | 76.6 | 17.5 | 22.5 |  |  | 629.4 |
|  |  |  | 7.4 |  | 3.8 | 7.3 | 21.5 | 5 | 7.8 |  |  | 266.8 |
|  | Healthy controls | 77 | 26.6 | 59.7% |  |  |  |  |  |  |  |  |
|  |  |  | 8.6 |  |  |  |  |  |  |  |  |  |
| UNIMAAS | Patients with schizophrenia | 34 | 27.9 | 35.3% | 20.9 | 7.2 | 23.1 | 12 | 11.8 |  |  |  |
|  |  |  | 5.4 |  | 6.2 | 5.6 | 7 | 5.1 | 5.6 |  |  |  |
|  | Healthy controls | 39 | 28.1 | 30.8% |  |  |  |  |  |  |  |  |
|  |  |  | 7.5 |  |  |  |  |  |  |  |  |  |
| UPENN | Patients with schizophrenia | 177 | 38.9 | 40.7% | 20.7 | 17.3 |  |  |  | 18.3 | 23.7 | 481.6 |
|  |  |  | 12.1 |  | 5.6 | 11.5 |  |  |  | 17 | 13.5 | 429.9 |
|  | Healthy controls | 193 | 36.4 | 53.4% |  |  |  |  |  |  |  |  |
|  |  |  | 14 |  |  |  |  |  |  |  |  |  |
| Zurich | Patients with schizophrenia | 60 | 30.5 | 25.0% |  |  |  |  |  |  |  |  |
|  |  |  | 8.5 |  |  |  |  |  |  |  |  |  |
|  | Healthy controls | 28 | 32.5 | 35.7% |  |  |  |  |  |  |  |  |
|  |  |  | 9.3 |  |  |  |  |  |  |  |  |  |

CDE: chlorpromazine dose equivalent; PANSS: Positive and Negative Syndrome Scale; SANS: Scale for the Assessment of Negative Symptoms; SAPS: Scale for the Assessment of Positive Symptoms; SD: standard deviation.

**Supplementary Figure S1.** Hedges’ *g* and *p*-values of (single) ComBat mega-analysis and the separate ComBat mega-analyses for cortical thickness data, cortical surface area data and subcortical volume data in the comparison of ENIGMA brain data between 2,897 patients with schizophrenia and 3,141 healthy controls.


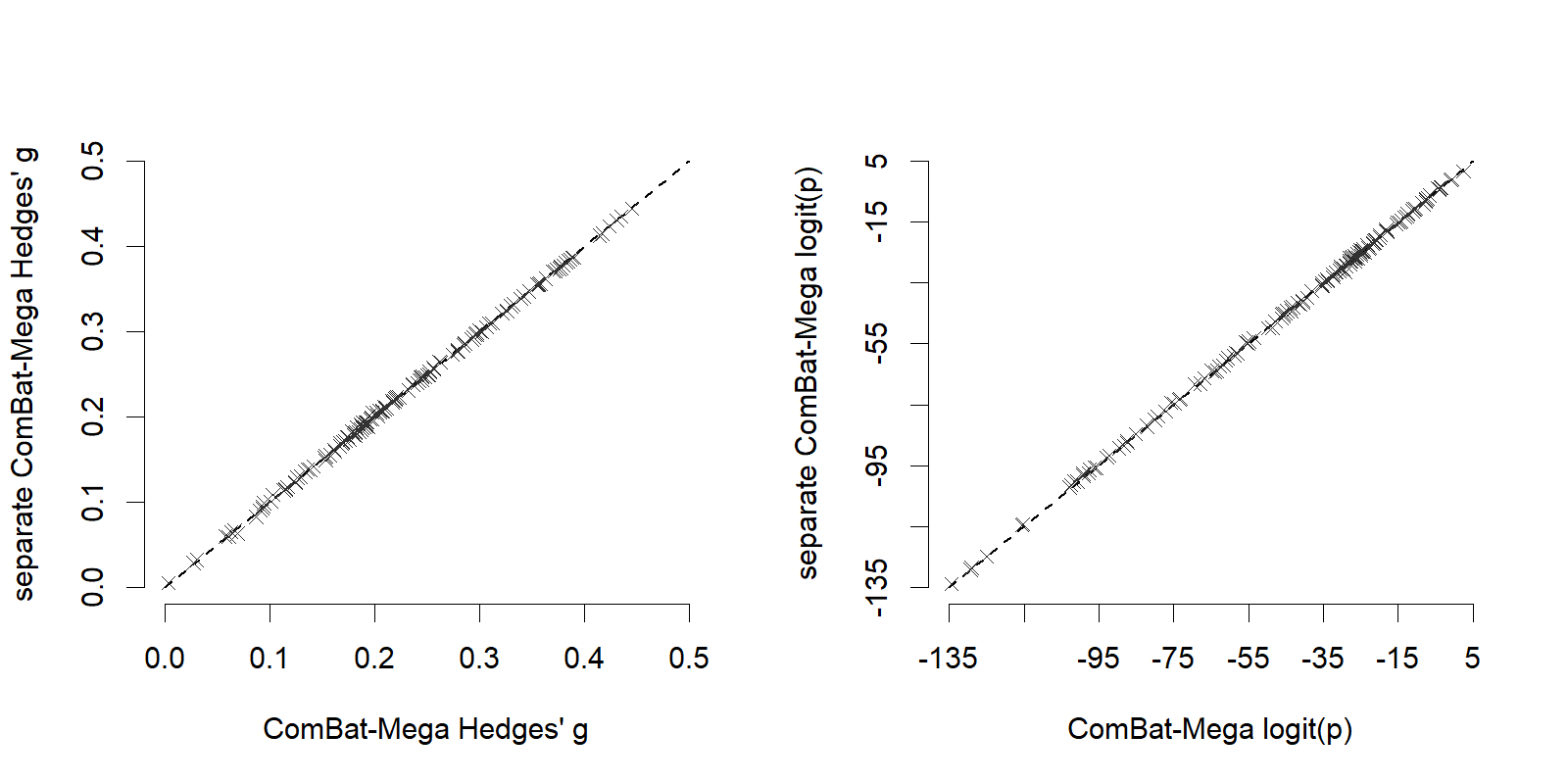


*Footnote:* Each cross represents an ROI. ComBat-Mega: (single) ComBat mega-analysis; separate ComBat-Mega: separate ComBat mega-analyses for cortical thickness data, cortical surface area data and subcortical volume data. The plots show that ComBat-Mega effect sizes and p-values are nearly identical to separate ComBat-Mega, as crosses are distributed along the diagonal lines.

**Supplementary Figure S2**. Hedges’ *g* and *p*-values of ComBat mega-analysis, mixed-effects mega-analysis and random-effect meta-analysis in the comparison of ENIGMA brain data between 2,897 patients with schizophrenia and 3,141 healthy controls, covarying by intracranial volume.


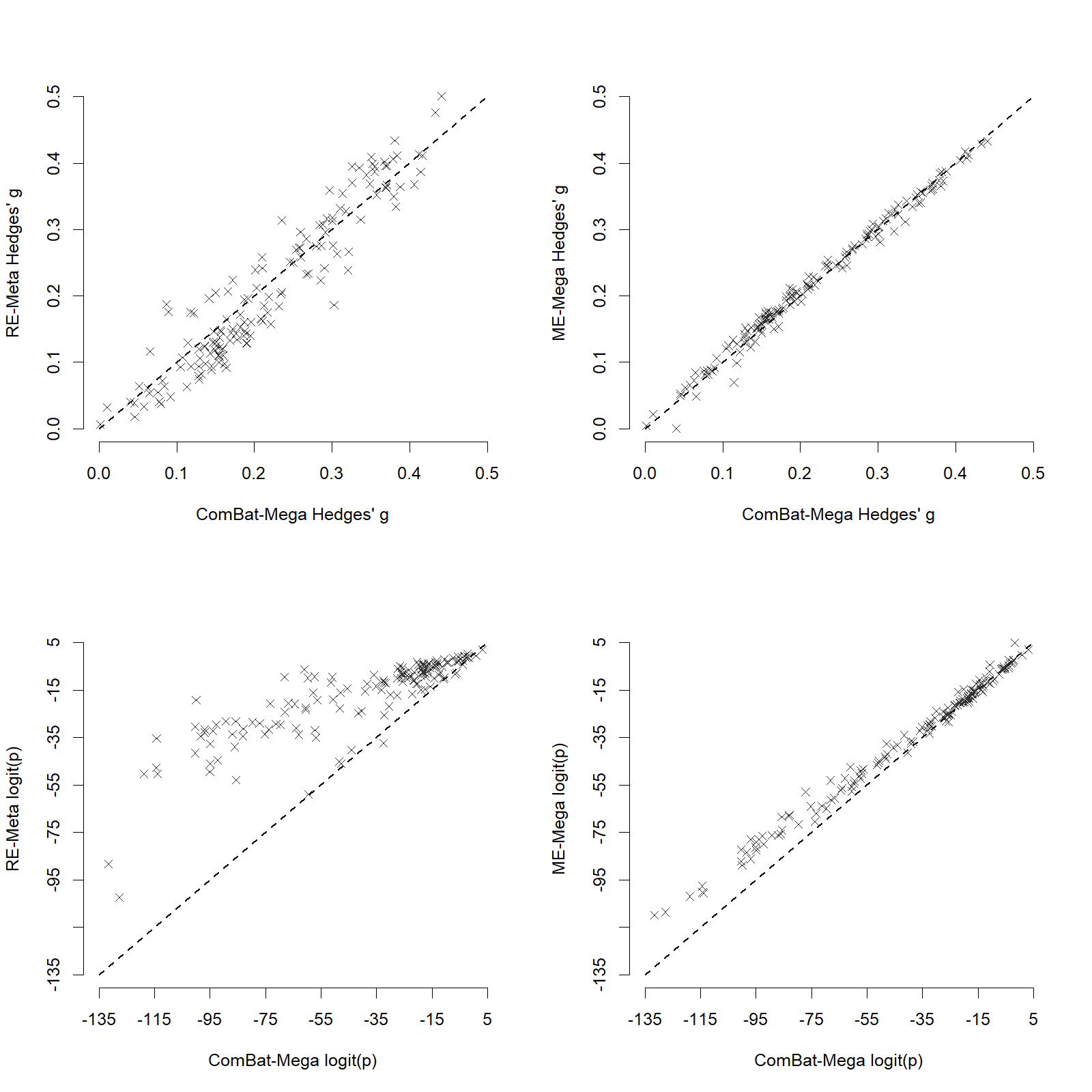


*Footnote:* Each cross represents an ROI. ComBat-Mega: ComBat mega-analysis; ME-Mega: mixed-effects mega-analysis; RE-Meta: random-effects meta-analysis. The top plots show that ComBat-Mega effect sizes are similar to ME-Mega and RE-Meta effect sizes, as crosses are mostly distributed around the diagonal lines. The bottom plots show that ComBat-Mega *p*-values are slightly smaller than ME-Mega *p*-values (crosses tend to be slightly above the diagonal line) and substantially smaller than RE-Meta *p*-values (crosses are clearly above the diagonal line).
